# Tailoring Cu-Based Catalysts Supported on ZrO_2_–Al_2_O_3_ for Efficient and Selective Ethanol Conversion to Ethyl Acetate

**DOI:** 10.1021/acsmaterialsau.5c00017

**Published:** 2025-04-10

**Authors:** Isabel C. Freitas, Davi D. Petrolini, Jean Marcel R. Gallo, Paula C. P. Caldas, Daniela C. de Oliveira, João B. O. Santos, Clelia Mara de Paula Marques, José Maria Correa Bueno

**Affiliations:** † Departamento de Química, Universidade Federal de São Carlos, C.P. 676, 13565-905 São Carlos, São Paulo, Brazil; ‡ Departamento de Engenharia Química, Universidade Federal de São Carlos, C.P. 676, 13565-905 São Carlos, São Paulo, Brazil; § Laboratório Nacional de Luz Síncrotron, C.P. 6192, 13083-970 Campinas, São Paulo, Brazil

**Keywords:** ethanol valorization, ethyl acetate, Cu^+^/Cu^0^ ratio, ZrO_2_−Al_2_O_3_ support, metal–oxide interface

## Abstract

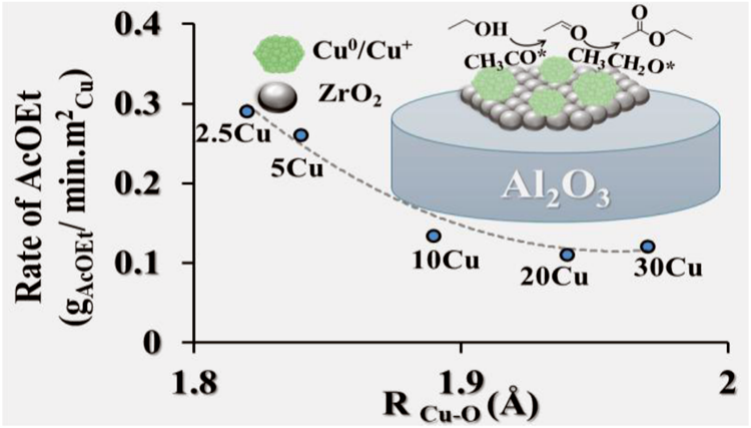

Selective conversion of ethanol to ethyl acetate is of significant industrial and environmental relevance, providing a sustainable route for adding value to ethanol. This study investigated Cu-based catalysts supported on ZrO_2_–Al_2_O_3_, focusing on the relationships among copper loading, metal–oxide interactions, and catalytic performance. Systematic variation of the copper content revealed that the Cu^+^/Cu^0^ ratio and the particle size distribution are crucial determinants of product selectivity. Lower copper loadings favored acetaldehyde production due to a higher Cu^+^/Cu^0^ ratio, while higher loadings favored Cu^0^ species and enhanced ethyl acetate selectivity by facilitating the formation of acyl species at the metal–oxide interface. The incorporation of ZrO_2_ was important for the creation of active sites necessary for condensation reactions. Advanced characterization techniques ((diffuse reflectance infrared Fourier transform spectroscopy DRIFTS)-CO, X-ray photoelectron spectroscopy (XPS), and extended X-ray absorption fine structure (EXAFS)) elucidated key electronic and structural properties, showing the need to tailor the copper loading and the composition of the support to ensure efficient and sustainable ethanol conversion.

## 1 Introduction

The development of innovative technologies that can combine efficiency with environmental sustainability has become a global priority, attracting the attention of researchers, industries, and governments. One of the most promising strategies is the use of ethanol, a renewable resource derived from the fermentation of cellulosic waste. This versatile compound has excellent potential as a key material for the chemical industry^1,2^ since alcohol chemistry can be used to manufacture a diverse portfolio of value-added products including ethyl acetate, acetaldehyde, and hydrogen.

Ethyl acetate, obtained by ethanol dehydrogenation, is a solvent widely used in the paint, adhesive, and coating industries. It is increasingly preferred to traditional solvents due to its lower toxicity and reduced environmental footprint than carbonylated compounds, such as acetone, methyl ethyl ketone (MEK), and methyl isobutyl ketone, and aromatic solvents, including xylene and toluene.^3−5^

A one-pot synthesis of ethyl acetate from ethanol can be performed according to two possible routes: the oxidative process^6,7^ or the dehydrogenative process.^3,7−10^ In the oxidative process, where the primary products are acetaldehyde, acetic acid, and CO_2_, the efficiency can be limited by the flammability range of ethanol/O_2_ mixtures, requiring careful control of the reaction conditions to ensure safety.^11−13^ In contrast, the dehydrogenative process is simpler, noncorrosive, less toxic, and requires only ethanol as a feedstock, with the added benefit of coproduction of hydrogen for use in subsequent hydrogenation reactions.^3,4,14^

Various heterogeneous catalysts have been employed for the selective conversion of ethanol, although copper-based catalysts have emerged as particularly effective for the dehydrogenative synthesis of ethyl acetate and acetaldehyde.^3,6−10,15−19^ Notably, several studies have found that the selection of a suitable support material can be crucial for controlling the formation of byproducts during this reaction. Iwasa and Takezawa^8^ investigated the use of copper catalysts supported on materials, including SiO_2_, ZrO_2_, Al_2_O_3_, MgO, and ZnO, for the direct conversion of ethanol. It was found that for Cu/SiO_2_ catalysts, the condensation reaction between ethanol and acetaldehyde to form ethyl acetate predominantly occurred on the copper surface, while for Cu/ZrO_2_ systems, the reaction took place on the support surface.

Previous work investigating the effect of the support on the active sites of Cu-based catalysts^17,20^ in the selective conversion of ethanol found that the Cu/SiO_2_ catalyst presented high selectivity toward acetaldehyde, while the Cu/ZrO_2_ catalyst exhibited high selectivity toward ethyl acetate. However, the activity of the Cu/ZrO_2_ catalyst was strongly dependent on the ZrO_2_ phase of the support, with the monoclinic phase being the most significant contributor to the high selectivity of the Cu/ZrO_2_ catalyst.^18^

Building on these findings, Cu-based catalysts, particularly CuCeZr solid solutions, have significantly improved ethyl acetate selectivity and ethanol conversion, with high long-term stability over 1000 h.^21^ Similarly, catalysts based on amorphous zirconia-supported copper have been found to enhance ethyl acetate yield by optimizing the acid–base site distribution and reducing side reactions that produce C_4_ byproducts.^22^ Furthermore, Ni-based catalysts modified with indium have been explored as a nontoxic alternative to CuCr catalysts, achieving high ethyl acetate selectivity by inhibiting undesired condensation reactions.^23^ Another approach involves the use of Mg–Fe mixed oxide catalysts doped with transition metals (Ni, Cu, Co, Mn, or Cr), where Cu and Ni enhancements improved ethyl acetate selectivity and ethanol conversion, showcasing long-term catalytic stability.^24^

Inui et al.^3^ proposed that the synthesis of ethyl acetate from ethanol over Cu–Zn–Zr–Al catalysts involves the formation of a hemiacetal intermediate in the reaction between acetaldehyde and ethanol, which occurs at active sites on the oxide surface, rather than the metal surface. Nevertheless, it remains unclear whether acetaldehyde/acyl or ethanol/ethoxy species are primarily involved in this mechanism. Gaspar et al.^6^ suggested that species could be transferred using a spillover mechanism, enabling the condensation reaction. Experiments using a physical mixture of catalyst and support (Cu/ZnO/Al_2_O_3_ + ZrO_2_) indicated that ethanol was initially converted to acetaldehyde on the Cu/ZnO/Al_2_O_3_ catalyst, followed by migration of the acetaldehyde to the ZrO_2_ surface, where it reacted with ethanol or ethoxy species to form the hemiacetal.

A strong copper–zirconia interface has been recognized as a critical factor in the development of Cu-based catalysts that are highly active, selective, and stable under reaction conditions.^25^ However, zirconia has a lower surface area than other oxides, such as Al_2_O_3_. Considering the effectiveness of Cu-based catalysts supported on Al_2_O_3_ and ZrO_2_ for the selective conversion of ethanol,^8,26^ an attractive approach is to combine the advantageous properties of these two supports. The use of ZrO_2_/Al_2_O_3_ as a support can provide multiple benefits, including the high surface area provided by alumina and the thermal stability of zirconia. Furthermore, zirconia presents both acidic and basic sites on its surface, which can enhance catalytic performance.^26,27^ Many studies have highlighted the versatility of ZrO_2_–Al_2_O_3_ as a support in different catalytic applications.^26,28−31^ Sagar et al.^26^ reported that Cu supported on ZrO_2_–Al_2_O_3_ exhibited high dispersion, forming two distinct types of Cu species on the catalyst surface. Freitas et al.^19^ demonstrated that the catalytic properties of Cu/ZrO_2_ catalysts in the selective conversion of ethanol were closely related to the specific electron density of the supported Cu species, influenced by particle size and the metal–oxide interface.

Despite these advancements, uncertainties remain concerning the key physicochemical properties governing the behavior of oxides in the one-pot synthesis of ethyl acetate. The species involved in this reaction and the role of the metal–oxide interface are still unclear. Therefore, this study aimed to elucidate the influence of electronic properties of the metal by varying the degree of dispersion of the active phase on the support. Investigation was also made of the structural properties of the support in Cu/ZrO_2_/Al_2_O_3_ catalysts, focusing on the generation of sites highly selective for ethyl acetate formation.

Elucidation of the intricate physicochemical properties of the samples employed a comprehensive suite of characterization techniques, including N_2_ adsorption–desorption for surface area and porosity, N_2_O titration for metal dispersion, and X-ray diffraction (XRD) for crystalline structure and phase purity. X-ray photoelectron spectroscopy (XPS) was used to analyze surface composition and oxidation states, while diffuse reflectance infrared Fourier transform spectroscopy (DRIFTS) was used to study CO adsorption and the active surface sites. Temperature-programmed reduction (TPR) assessed catalyst reducibility and metal–support interactions. Extended X-ray absorption fine structure (EXAFS) provided insights into the local atomic structure, coordination, and bonding. This multifaceted approach not only ensured a thorough understanding of the structural and electronic properties of the catalyst but also provided a basis for correlating these characteristics with catalytic activity and selectivity, ultimately guiding the design of more efficient catalytic systems.

## 2 Experimental Section

### 2.1 Sample Preparation

The γ-Al_2_O_3_ support was prepared by the sol–gel method.^32^ First, aluminum-trisec-butoxide (ATSB) (Aldrich) was added to a round-bottomed flask containing 2-butanol under vigorous stirring and refluxing at 358 K for 1 h. After this period, ATSB was complexed with 1,3-butanediol, maintaining stirring for further 2 h. Finally, deionized water was added to complete the hydrolysis step, and the solution was agitated for 1 h. The molar ratio of the reactants was 1:8.5:4.5:12 for ATSB/2-butanol/1,3-butanediol/H_2_O. After removal of the solvent by evacuation, the gel was dried at 318 K, under vacuum, using a rotary evaporator. The resulting gel was subjected to further drying overnight at 373 K in an oven. The support was then calcined under a flow of synthetic air at 100 mL/min, with the temperature increased from room temperature to 823 K at a rate of 10 K/min, maintaining the final temperature for 4 h. Incipient wetness impregnation of the γ-Al_2_O_3_ support was then performed using a solution of zirconium(IV) oxynitrate hydrate (99.9%, Aldrich) to obtain a ZrO_2_ loading of 30 wt %. The sample was calcined under a flow of synthetic air at 120 mL/min, with the temperature increased from room temperature to 773 K at a rate of 10 K/min, maintaining the final temperature for 5 h. The resulting ZrO_2_/γ-Al_2_O_3_ support was denoted 30Zr/Al. Tetragonal ZrO_2_ (commercial grade, Saint-Gobain NorPro) was also used as a support, labeled as *t*-ZrO_2_, and after copper impregnation, the resulting catalyst was referred to as Cu/*t*-Zr.

Catalysts with Cu supported on the materials described above were prepared by incipient wetness impregnation of the supports with a solution of Cu(NO_3_)_2_·2.5H_2_O (98%, Aldrich) in methanol. The impregnated solids were dried at 383 K and then calcined at 673 K in a flow of air for 12 and 5 h, respectively. The samples were denoted *x*Cu/30Zr/Al, where *x* represents the theoretical Cu loading (2.5, 5, 10, 20, 30, and 50 wt %).

### 2.2 Characterization

The specific surface area (*S*_BET_) and pore volume (*V*_p_) of the samples were determined using nitrogen adsorption–desorption isotherms obtained at 77 K using a Quantachrome Nova 1200 instrument. Prior to the measurements, the samples were degassed for 2 h at 423 K under vacuum.

To identify the structures of the samples, X-ray diffractograms of the calcined materials were recorded using a Rigaku DMAX 2500 diffractometer operating at 40 kV and 40 mA, with Cu Kα radiation (λ = 1.54060 Å) in the 2θ range from 5 to 85°, with a step size of 0.020° and a step scan time of 10.0 s. Phase identification was achieved by comparison with JCPDS database cards.

XPS data for the reduced samples were obtained using a spectrometer (PHOIBOS 150-HSA 3500, 9 channeltrons, SPECS GmbH) equipped with an Al Kα source (*E* = 1486.6 eV) operating at 15 kV, with an *E*_pass_ value of 40 eV, energy step of 0.6 eV, and acquisition time of 2 s per point. The samples were placed on stainless steel sample holders and transferred under an inert atmosphere to the XPS prechamber, where they remained for 12 h in a vacuum environment. The residual pressure inside the analysis chamber was less than 1 × 10^–9^ Torr. The binding energies (BE) of Cu 2p, Zr 3d, Al 2p, O 1s, Cu LMM, and C 1s were referenced to the C 1s peak at 284.5 eV, ensuring an accuracy of ±0.2 eV. All of the samples were reduced in a furnace, with heating at a rate of 10 K/min from room temperature to the desired temperature based on the H_2_-TPR behavior. The samples were held at 573 K for 30 min, under a 200 mL/min flow of 5% H_2_/He, followed by cooling to room temperature before subsequent analysis. X-ray-induced Auger electron spectra of the reduced samples were also obtained in the kinetic energy region of 928–902 eV. The modified Auger parameter (α_Cu_) was calculated according to eq 1(33)
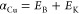
1where *E*_B_ is the binding energy of the Cu 2p core level and *E*_K_ is the kinetic energy of the Cu LMM Auger electron.

TPR profiles were recorded using a Micromeritics Pulse Chemisorb 2705 instrument equipped with a thermal conductivity detector (TCD). To remove surface contaminants, the sample (0.04 g) was loaded into a quartz reactor and pretreated for 1 h at 423 K in a flow of He. After cooling to room temperature, a flow of 5% H_2_/N_2_ (30 mL/min) was passed through the sample, and the temperature was increased to 773 K at a rate of 10 K/min while recording the TCD signal. A cooling trap placed between the sample and the detector was used to retain the water formed during the reduction process.

Using the same TPR apparatus described above, N_2_O titration was performed to determine the surface area of the dispersed metallic Cu. This procedure consisted of two steps: (i) oxidation of Cu^0^ to Cu_2_O using N_2_O as an oxidation agent and (ii) temperature-programmed reduction of surface Cu_2_O species. The Cu/ZrO_2_/Al_2_O_3_ samples were prereduced up to 523 K, followed by cooling to 303 K and exposure to 1% N_2_O/He (30 mL/min) for 10 min. The second H_2_-TPR step was performed by increasing the temperature to 773 K at a rate of 10 K/min under 5% H_2_/He. The consumption of hydrogen was used to calculate the amount of oxygen deposited after N_2_O titration. A total of 1.46 × 10^19^ Cu atoms/m^2^ and a stoichiometry of 2Cu/H_2_ were used.^34^ The Cu surface area (SA_Cu_) was calculated based on the following relationship
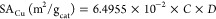
2where *C* is the Cu content (%) and *D* is the metallic copper (Cu^0^) dispersion (%).

Assuming a spherical shape, the average copper metal particle size (*d*_Cu_) was determined using the following expression^35^
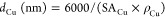
3where ρ_Cu_ is the copper density (8.92 g/cm^3^).

Diffuse reflectance infrared Fourier transform spectroscopy (DRIFTS) analysis of CO adsorption on the reduced Cu/ZrO_2_/Al_2_O_3_ catalysts was performed using a Thermo Nicolet 4700 NEXUS FT-IR spectrophotometer equipped with an MCT detector and a reactor cell (Spectra Tech) with CaF_2_ windows (DRIFT HTHV cell). FT-IR spectra were acquired using 64 scans at a resolution of 4 cm^–1^. Before reduction, the samples were dried for 1 h at 473 K under a flow of He. The catalysts were then reduced for 2 h at 573 K in a 25% H_2_/He mixture. CO was introduced for 1 s and adsorbed at 323 K using a CO pressure of 20 Torr. Temperature-programmed desorption (TPD) of adsorbed CO was performed in a flow of He (30 mL/min), with heating at a rate of 10 K/min.

EXAFS spectra were obtained at the D08B-XAFS 2 beamline of the Brazilian National Synchrotron Light Laboratory (LNLS), which was equipped with a Si(111) double crystal monochromator. The Cu/ZrO_2_/Al_2_O_3_ samples were prepared as self-supporting pellets consisting of a mixture of the catalyst and boron nitride, which were placed in a tubular quartz furnace, sealed with Kapton-refrigerated windows, for transmission measurements. Spectra for the samples reduced in H_2_ at 523 K were acquired at 473 K. The EXAFS signal was processed using the Athena and Artemis software packages. The fits were performed by fixing the same passive electron amplitude reduction factor (*S*_0_^2^) and photoelectron energy origin correction (Δ*E*_0_) determined for the reference compounds. The adjustable parameters were the coordination number of the path (*N_i_*), the effective half-path length (*R_i_*, equal to the interatomic distance for single-scattering paths), and the Debye–Waller factor (σ^2^*_i_*). The local environment of the Cu atoms was determined from the EXAFS data using the phase shift and amplitude functions for the Cu–Cu and Cu–O interactions, calculated considering multiple scattering processes, employing FEFF v. 6.0 software. The oscillations were weighted with *k*^2^ and Fourier-transformed within the *k* range from 3.5 to 12 Å^–1^. A relatively narrow fitting range (Δ*k*) was used in this study because the Hf L3-edge (9651 eV) from HfO_2_ impurities (present in the ZrO_2_ support) interfered with the extraction of the Cu K-edge oscillations at higher *k* values. Based on the EXAFS data and assuming a cuboctahedral model for copper particles,^36^ the Cu^0^ surface area was calculated using eq 2 in combination with the following relationship
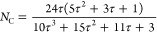
4where *N*_C_ is the coordination number of the Cu cluster and τ is the copper cluster order.

From the cluster geometry, characteristics of the cuboctahedral particles, such as the total number of atoms and particle size, could be obtained using the following equations
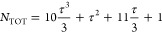
5where *N*_TOT_ is the total number of atoms and τ is the order of the cluster.
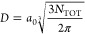
6where *a*_0_ is the lattice parameter calculated based on the bond distance for each sample and *N*_TOT_ is the total number of atoms.

### 2.3 Catalytic Test Reaction

The activity and selectivity of the catalysts in ethanol conversion were evaluated using a continuous-flow tubular fixed-bed quartz reactor (10 mm i.d.) containing 0.2 g of catalyst. The reactions were performed in the temperature range from 473 to 548 K at atmospheric pressure. Gas phase ethanol (99.9%) was introduced into the catalytic reactor by passing helium (He) through a saturator submerged in a water bath maintained at 328 K, ensuring an ethanol partial pressure of 37.4 kPa. The reaction was performed with a contact time of 38 min, determined using *W*/*F*, where *W* is the catalyst weight (g) and *F* is the ethanol flow rate (g/min). The catalyst samples were reduced in situ in a flow of pure hydrogen, with heating from room temperature to 523 K at 10 K/min and maintaining the final temperature for 90 min. Gas phase ethanol (99.9%) was introduced using helium at a flow rate of 10 mL/min. The reaction products were periodically collected for analysis by online gas chromatography using a Varian GC-3400 CX instrument fitted with a Chromosorb 102 column. Ethanol conversion and product selectivity were calculated on a carbon basis.

## 3 Results

### 3.1 Textural Properties and Structures

**Figure 1:**
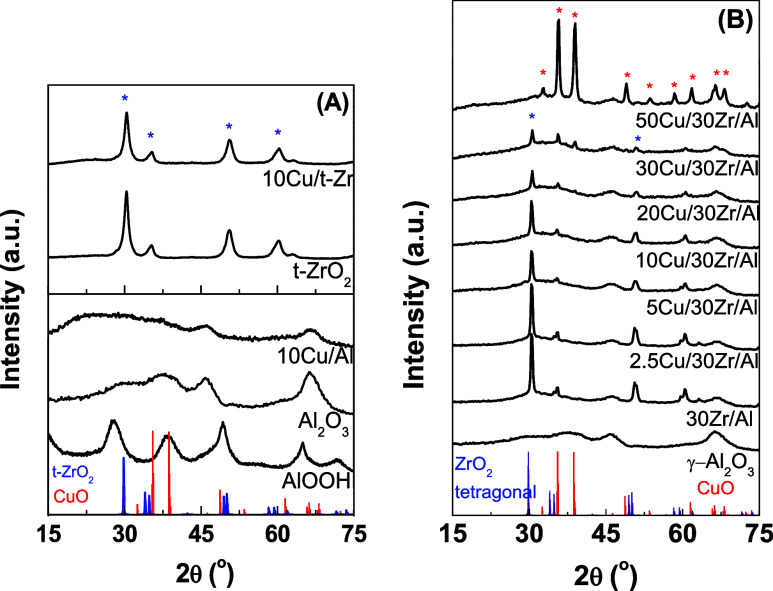
X-ray diffractograms of (A) calcined Cu/Al and Cu/t-Zr samples and (B) Cu/30Zr/Al samples with different Cu contents.

Figure 1 presents the X-ray diffractograms of the 10Cu/t-Zr and 10Cu/Al samples (A) and *x*Cu/30Zr/Al samples with varying Cu contents (B). The diffractograms of pure ZrO_2_ and pure Al_2_O_3_ are also included for comparison. The sol–gel method employed for alumina preparation resulted in the formation of a pseudoboehmite phase (Figure 1A), consistent with the previous findings by Dumeignil et al.^32^ Treatment of the Al_2_O_3_ precursor at 773 K caused dehydroxylation of the pseudoboehmite, resulting in a diffractogram characteristic of a weakly crystalline γ-Al_2_O_3_ phase.

As shown in Figure 1A, the addition of 10 wt % Cu to the supports did not lead to any diffraction peaks corresponding to the crystalline CuO phase, indicating that copper was highly dispersed on the catalyst surface. After deposition of ZrO_2_ on the Al_2_O_3_ surface, diffraction peaks at 2θ values of 30.3, 35.2, 50.6, and 60.2° could be attributed to the tetragonal zirconia phase (JCPDS No. 17-0923) (Figure 1B). However, increasing the copper content on the 30Zr/Al support resulted in decreased intensity of the tetragonal zirconia diffraction peaks. This could be explained by greater X-ray absorption caused by the higher copper content since Cu Kα radiation was used as the X-ray source.

The diffractogram for bulk CuO exhibited sharp peaks at 2θ values of 32.6, 35.6, 38.6, 48.8, 53.6, 61.6, 66.4, 68.1, 72.3, and 75.1°, corresponding to the crystalline CuO phase with a tenorite structure (JCPDS No. 48-1548) (Figure 1B). The diffractograms of the *x*Cu/30Zr/Al samples with Cu contents of up to 20 wt % did not show peaks characteristic of CuO species, suggesting that the CuO crystallites were below the detection limit of the XRD technique, probably existing as small crystallites with diameters smaller than 4 nm. However, when the Cu content exceeded 20 wt %, characteristic CuO peaks appeared at 2θ values of 35.4, 38.7, 48.7, 58.3, 61.5, 66.1, and 68.1°, together with the 30Zr/Al peaks (Figure 1B), indicating the agglomeration of copper oxide species.

The XRD peaks corresponding to supported CuO were the most intense for the 50Cu/30Zr/Al sample, suggesting increased crystallinity and the formation of bulk CuO at higher Cu loadings (≥20 wt %). Additionally, the diffractograms (Figure 1B) did not show any peaks characteristic of mixed oxides formed between CuO and the 30Zr/Al support.

Some textural characteristics of the supports and the *x*Cu/30Zr/Al samples with varying Cu contents are listed in Table 1, together with the metallic Cu surface area (SA_Cu_), the apparent metallic Cu dispersion (*D*_Cu_), and the metal particle size measured by N_2_O titration.

**Table 1:** Textural Properties of the Supports and the *x*Cu/30Zr/Al Samples with Different Cu Contents^a^

sample	*S*_BET_ (m^2^/g_cat_)	*S*_BET_ (m^2^/g_Al_2_O_3__)	*S*_BET_ (m^2^/g_30Zr/Al_)	*V*_p_ (cm^3^/g_cat_)	*D*_p_ (nm)	*D*_Cu_ (%)	SA_Cu_ (m_Cu_^2^/g_cat_)
Al_2_O_3_	370 (±0.5)	370	--	0.65	6.0	--	--
30ZrO_2_/Al_2_O_3_	313 (±0.5)	447	--	0.43	5.0	--	--
2.5Cu/30Zr/Al	275 (±0.4)	--	282	0.43	5.0	68	11
5Cu/30Zr/Al	269 (±0.8)	--	283	0.43	4.7	88	28
10Cu/30Zr/Al	242 (±0.9)	--	269	0.35	5.2	79	54
20Cu/30Zr/Al	221 (±1.2)	--	276	0.33	4.8	74	99
30Cu/30Zr/Al	202 (±0.6)	--	288	0.23	5.0	52	101
50Cu/30Zr/Al	180 (±0.9)	--	360	0.24	4.8	30	97
10Cu/Al	315 (±0.4)	--	--	0.56	6.1	89	45
t-ZrO_2_	152 (±1.0)	--	--	0.18	4.4	--	--
10Cu/t-Zr	119 (±1.2)	--	--	0.15	4.6	56	36

a Also shown are the values for the metallic Cu surface area (SA_Cu_), the apparent metallic Cu dispersion (*D*_Cu_), and the metal particle size measured by N_2_O titration (*D*_p_).

The *S*_BET_ surface area of the pure Al_2_O_3_ was 370 m^2^/g, with a decrease to 313 m^2^/g after impregnation of the alumina with 30 wt % ZrO_2_. When the results were expressed relative to the alumina support (m^2^/g_Al_2_O_3__), it could be seen that a contribution from ZrO_2_ increased the apparent surface area of the alumina by 77 m^2^/g_Al_2_O_3__. This increase suggested that a significant portion of the ZrO_2_ was segregated on the alumina surface, as further evidenced by XRD analysis.

The *S*_BET_ and pore volume (*V*_p_) values for the calcined *x*Cu/30Zr/Al samples were generally lower than for the pure supports. For the samples with lower Cu contents (up to 20 wt %), the increase of the surface area could be attributed to the consumption of hydroxyl groups on the surface of the support by reactions with the copper oxide precursor. However, the surface area decreased as the Cu content increased above 20 wt %, probably due to the agglomeration of CuO species and the covering of the support pores by the active phase.

The N_2_O titration data for the *x*Cu/30Zr/Al series (Table 1) showed that the highest apparent Cu^0^ dispersion was for the 5Cu/30Zr/Al catalyst. The dispersion of Cu^0^ decreased with increasing Cu content due to the progressive covering of the support surface by the active phase. Interestingly, an opposite trend was observed for the metallic Cu surface area, where an increase with higher Cu content could be attributed to the strong interaction between the copper oxide species and the support surface.

**Figure 2:**
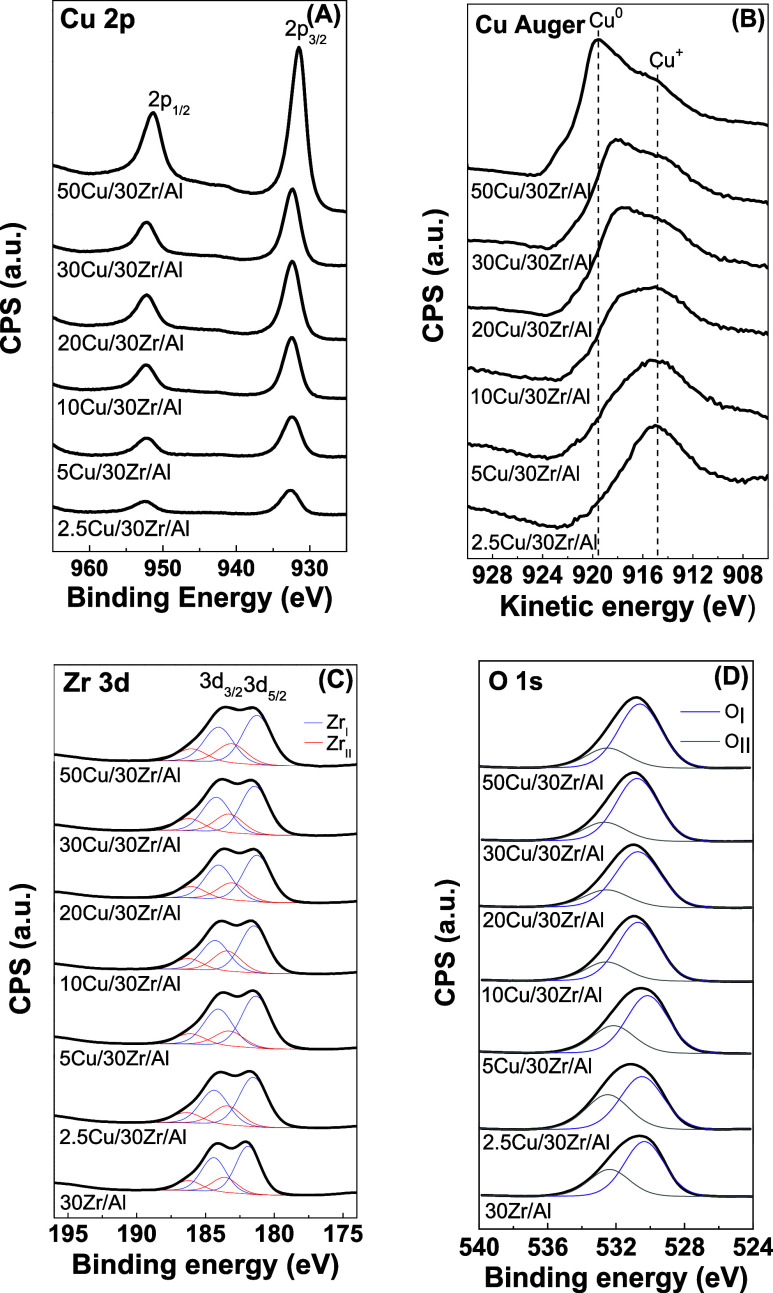
XPS core-level spectra of Cu 2p (A), Cu LMM Auger (B), Zr 3d (C), and O 1s (D) for the reduced *x*Cu/30Zr/Al samples. The deconvoluted Cu LMM Auger spectra and the corresponding surface Cu^0^ and Cu^+^ species are provided in Figure S1 and Table S1.

The XPS core-level spectra of Cu 2p, Cu LMM Auger, Zr 3d, and O 1s for the reduced *x*Cu/30Zr/Al samples are shown in Figure 2A–D, respectively. The XPS parameters are summarized in Table 2.

**Table 2:** XPS Data for the Supports and *x*Cu/30Zr/Al Samples with Different Cu Contents

				Zr 3d_5/2_ (eV)			O 1s (eV)		
samples	Cu 2p_3/2_ (eV)	Cu Auger (eV)	α_Cu_ (eV)	Zr_I_ species	Zr_II_ species	Al 2p (eV)	O_I_ species	O_II_ species	Cu/(Zr + Al)
Al_2_O_3_	--	--	--	182.3 (86)^b^	183.8 (14)	--	530.5 (68)^b^	532.4 (32)	--
t-ZrO_2_	--	--	--	--	--	74.4	530.7 (77)	532.8 (23)	--
30Zr/Al	--	--	--	181.8 (72)	183.5 (28)	74.4	530.4 (64)	532.3 (40)	--
2.5Cu/30Zr/Al	932.8 (2.8)^a^	914.8	1847.6	181.8 (72)	183.5 (28)	74.5	530.4 (60)	532.3 (40)	0.07
5Cu/30Zr/Al	932.6 (2.8)	914.8	1847.4	181.7 (74)	183.4 (26)	74.4	530.2 (67)	532.1 (33)	0.11
10Cu/30Zr/Al	932.5 (2.6)	917.8/914.8	1850.3/1847.3	181.7 (72)	183.4 (28)	74.4	530.6 (74)	532.5 (26)	0.25
20Cu/30Zr/Al	932.4 (2.4)	917.9/914.8	1850.3/1847.2	181.6 (73)	183.3 (27)	74.3	530.6 (75)	532.5 (25)	0.33
30Cu/30Zr/Al	932.4 (2.4)	918.2/914.8	1850.6/1847.2	181.7 (72)	183.4 (28)	74.4	530.7 (77)	532.6 (23)	0.48
50Cu/30Zr/Al	931.7 (2.2)	919.5	1851.2	181.6 (72)	183.2 (28)	74.3	530.7 (77)	532.6 (23)	0.97

a Full width at half-maximum (fwhm).

b Percentage of Zr and O species.

The spectra of all of the reduced samples exhibited symmetric Cu 2p_3/2_ and Cu 2p_1/2_ main peaks (Figure 2A) with binding energy (BE) values at approximately 931.7–932.8 and 952.4 eV, respectively, and spin–orbit coupling energy of 20 eV. No shakeup satellites at ∼942 eV were detected, suggesting the absence of Cu^2+^ species.^37^ Notably, there was a slight decrease in the BE for Cu 2p_3/2_ with increasing Cu content, from 932.8 eV for 2.5Cu/30Zr/Al to 931.7 eV for 50Cu/30Zr/Al (Table 2), probably due to electron transfer from Cu to the support. The fwhm for Cu 2p_3/2_ ranged from 2.2 to 2.8 eV, indicating the presence of at least two distinct copper species in different chemical environments. Therefore, the possible presence of traces of Cu^+^ could not be excluded since the BE for Cu^+^ overlaps with that for Cu^0^ in the Cu 2p core-level spectrum.^37^

To differentiate between Cu^0^ and Cu^+^ species (which have similar BEs), Figure 2B shows the X-ray-induced Auger electron spectra for the reduced *x*Cu/30Zr/Al samples in the kinetic energy range from 930 to 906 eV. Clear differences in the L_3_M_45_M_45_ Auger spectra were due to specific bonding interactions at the metal–oxide interface. Peaks at 917.8–919.5 and 914.8 eV (Table 2) could be attributed to Cu^0^ and Cu_2_O (Cu^+^), respectively.^18,19,38^ For the samples with low Cu contents (2.5–5 wt % Cu), the kinetic energy (KE) was ∼914.8 eV. With increasing Cu content, the peak characteristic of metallic Cu shifted to higher KE values, reaching 919.5 eV for the 50Cu/30Zr/Al sample (Figure 2B and Table 2). The modified Auger parameter (α_Cu_) values were 1850.3–1851.2 eV for Cu^0^ and 1847.6 eV for Cu^+^, showing that Cu^+^ dominated at low Cu contents (<10 wt %), while Cu^0^ was more prevalent in samples with higher Cu contents (≥20 wt %). For intermediate Cu contents (10–20 wt %), equilibrium between Cu^0^ and Cu^+^ at the surface was observed (Figure 2B).

For all of the samples, the Zr 3d core-level spectrum exhibited a spin–orbit doublet, split into 3d_5/2_ and 3d_3/2_, with an energy gap of 2.4 eV between them and a relative intensity ratio (I 3d_5/2_/I 3d_3/2_) of 1.5 (Figure 2C). This indicated the presence of ZrO_2_-like species, consistent with literature data.^39^ Decomposition of the spectra revealed signals corresponding to two types of zirconium species. The first, denoted species I (Zr_I_), with a lower binding energy of approximately 181.6 eV, was characteristic of Zr^4+^ ions in pure ZrO_2_, in agreement with the reported value of 182.2 ± 0.2 eV.^27^ The second, species II (Zr_II_), with a higher binding energy of approximately 183.2 eV, could be attributed to zirconium species bound to a more electron-attractive environment or to the formation of partially reduced Zr^δ+^ sites.^39^ These electron-deficient Zr^δ+^ species are likely associated with the Cu–O–Zr interfacial regions, where electronic interactions between Cu and ZrO_2_ induce charge redistribution.^40^

Broad peaks in the O 1s XPS spectra (fwhm = 3.7 eV) were due to overlapping contributions of zirconia lattice oxygen and supported copper species. The O 1s peaks were decomposed into two components, as shown in Figure 2D, with the relative contributions shown in Table 2. According to the literature,^41−43^ there are two types of oxygen species in the *x*Cu/30Zr/Al system after reduction: lattice oxygen species from ZrO_2_, Al_2_O_3_, and/or Cu_2_O (O_I_) and hydroxyl species associated with Zr–OH and Al–OH (O_II_), with binding energies in the ranges of 530.4–530.7 and 532.1–532.8 eV, respectively (Table 2). It has been shown that oxygen species with BE ≥ 531.0 eV can be attributed to OH groups, chemisorbed oxygen, or carbonate species.^44^

The Al 2p binding energy of 74.4 ± 0.2 eV was the same for all of the *x*Cu/30Zr/Al samples and was identical to that of pure alumina (Table 2).^27^

The value of the XPS Cu/(Zr + Al) atomic ratio for the reduced *x*Cu/30Zr/Al samples increased with increasing copper content (Table 2), indicating Cu enrichment on the support surface, in agreement with the N_2_O titration data (Table 1). This suggested that the metallic particle size increased as the copper content increased. However, the particles were not entirely composed of Cu^0^ species but rather a combination of Cu^0^ and Cu^+^ species on the support surface.

**Figure 3:**
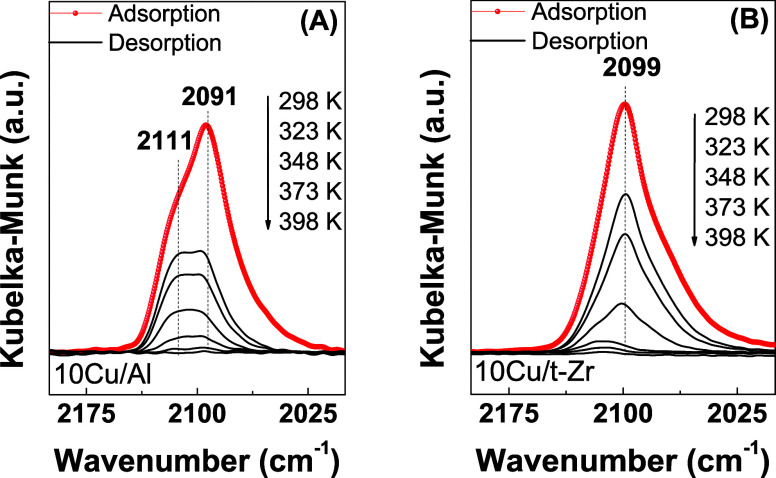
FT-IR spectra in the high-frequency region (2200–2000 cm^–1^) for CO adsorption on the reduced 10Cu/Al (A) and 10Cu/t-Zr (B) samples.

**Figure 4:**
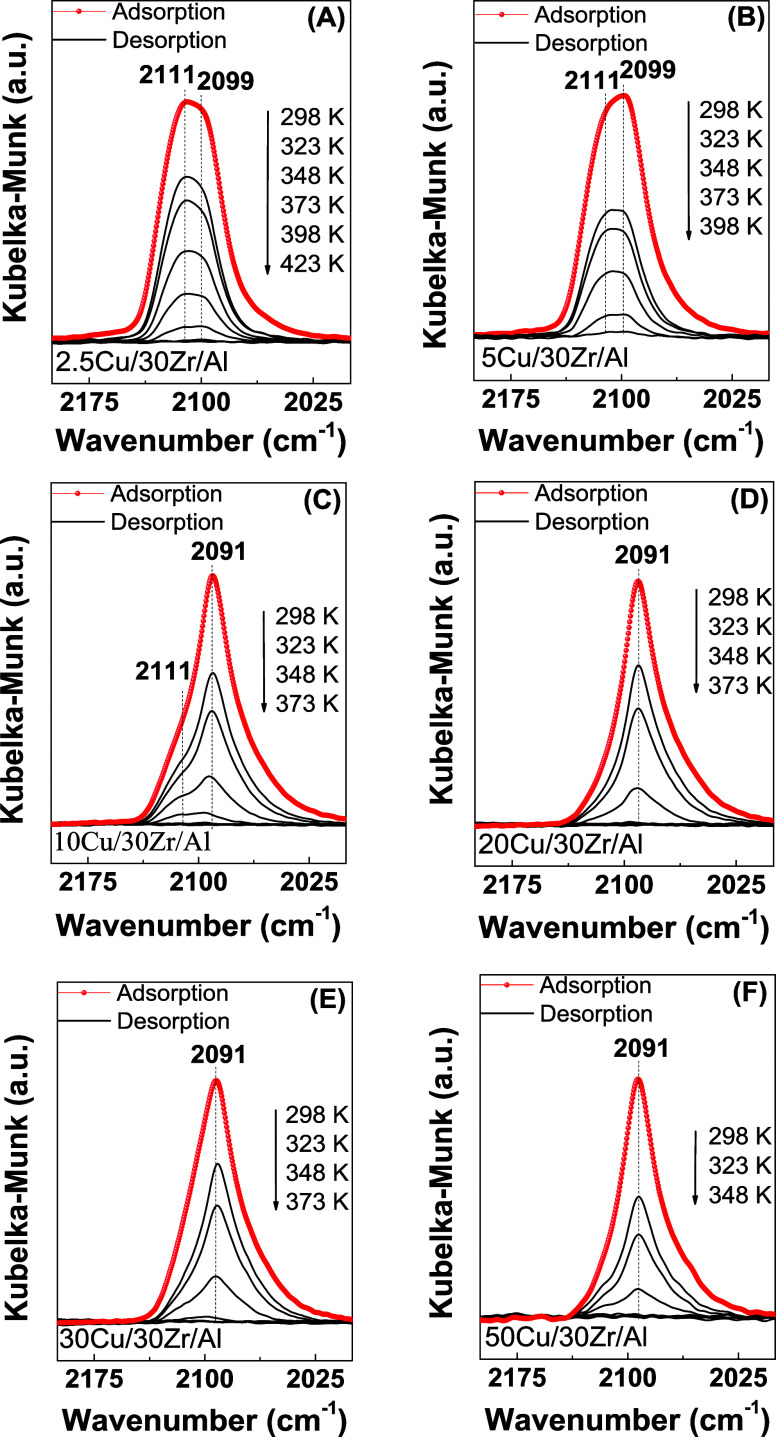
FT-IR spectra in the high-frequency region (2200–2000 cm^–1^) for CO adsorption on the reduced Cu/30Zr/Al samples: 2.5Cu/30Zr/Al (A), 5Cu/30Zr/Al (B), 10Cu/30Zr/Al (C), 20Cu/30Zr/Al (D), 30Cu/30Zr/Al (E), and 50Cu/30Zr/Al (F).

The DRIFTS spectra for CO adsorption on the reduced *x*Cu/30Zr/Al samples are shown in Figures 3A,B and 4A–F for the high-frequency (2200–2000 cm^–1^) and low-frequency (1800–1200 cm^–1^) regions, respectively, together with the temperature-programmed desorption (TPD) spectra of CO in the temperature range of 298–773 K. According to the literature,^45^ the adsorption of CO on copper surfaces results in IR bands in the 2250–2000 cm^–1^ spectral region, corresponding to linear or bridge bonding of CO species interacting with CuO, Cu_2_O, or Cu^0^ sites. Bands at 2116–2111 cm^–1^ have been attributed to Cu^+^–CO carbonyls, while bands at ≤2100 cm^–1^ have been assigned to CO adsorbed on Cu^0^.^45^

Analysis of the bands in the copper spectral region related to CO adsorption revealed that the IR spectra of the reduced 10Cu/Al, 10Cu/t-Zr, and *x*Cu/30Zr/Al samples presented strong bands with maxima at 2111, 2099, and 2091 cm^–1^, respectively (Figures 3A,B and 4A–F). According to Hadjiivanov and Vayssilov,^45^ Cu^0^–CO carbonyls can absorb at the same frequency as Cu^+^–CO when the copper is highly dispersed on the surface. Distinction between these two species can be achieved considering their different thermal stabilities since surface Cu^0^–CO carbonyls are easily destroyed when the temperature is increased, while Cu^+^ cations form surface carbonyls that are more thermally stable due to the stronger σ-bonds of Cu^+^–CO species.

The 10Cu/Al sample (Figure 3A) exhibited two absorption frequencies, at 2111 and 2091 cm^–1^, assigned to linear CO–Cu^+^ and CO–Cu^0^ species, respectively.^36,46,47^ In contrast, CO adsorption over the 10Cu/t-Zr catalyst (Figure 3B) resulted in a single band, assigned to CO linearly adsorbed on Cu^0^ sites.^18,19^ These features suggested that CO adsorption on the *x*Cu/30Zr/Al samples may have occurred at the surfaces of copper particles deposited on the alumina and/or zirconia since it is unlikely that ZrO_2_ completely covered the Al_2_O_3_ support. For the *x*Cu/30Zr/Al samples with lower copper contents (2.5 and 5 wt %), a broad band resulted from the overlap of three absorptions at frequencies of 2011, 2099, and 2091 cm^–1^. The bands at 2011 and 2091 cm^–1^ were probably associated with CO–Cu^+^ and CO–Cu^0^ on the surface of copper deposited on alumina, while the band at 2099 cm^–1^ corresponded to CO–Cu^0^ on the zirconia surface. However, the TPD-CO analysis performed after adsorption showed that higher desorption temperatures (423 and 398 K, respectively) were required compared to the other catalysts, indicating higher concentrations of Cu^+^ species on the surfaces of these catalysts.

As the copper content in the *x*Cu/30Zr/Al catalysts increased, the intensities of the bands at 2011 and 2099 cm^–1^ decreased, while the band at 2091 cm^–1^ became predominant, with high intensity (Figure 4C–F). The *x*Cu/30Zr/Al catalysts with copper contents above 5 wt % predominantly exhibited CO–Cu^0^ species adsorbed on copper particles, either on alumina or over large CuO particles that covered ZrO_2_ in contact with alumina. The predominance of Cu^0^ species was indicated by the low thermal stability observed in the TPD-CO measurements (at 273 and 348 K) for these catalysts compared to samples with lower copper contents (2.5 and 5 wt % Cu). A shoulder at 2111 cm^–1^, attributed to CO–Cu^+^ species, was observed for the catalysts with copper contents of up to 5 wt %. The intensity of this shoulder decreased with the increase of the Cu content. Hence, for the *x*Cu/30Zr/Al series, an increase of the copper content led to a lower Cu^+^/Cu^0^ ratio for the copper species on the surface, as evidenced by XPS.

According to Bianchi et al.,^48^ a portion of the carbon monoxide adsorbed on the copper surface in a Cu/ZrO_2_ catalyst can be transferred to the support surface by a spillover mechanism. Consequently, the carbonate and bicarbonate species observed in the 1800–1100 cm^–1^ spectral region may be formed in the reaction between CO activated on the surface of copper particles and oxygen ions and/or hydroxyl groups present on the support surface.^48^

**Figure 5:**
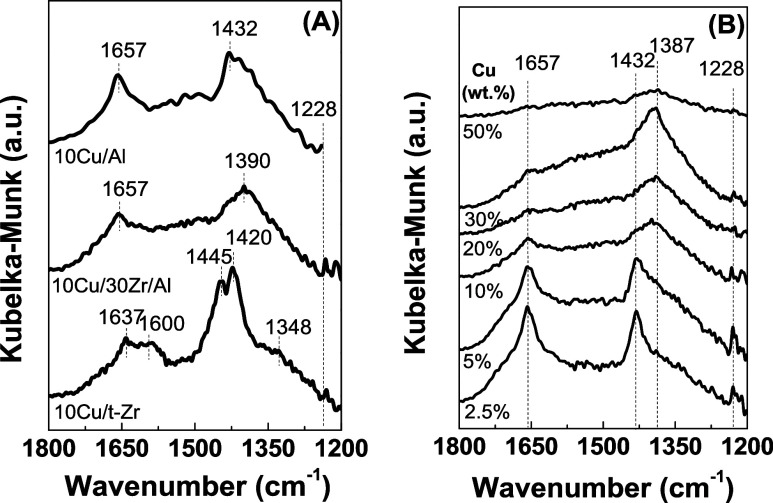
FT-IR spectra in the low-frequency region (1800–1200 cm^–1^) for CO adsorption on the reduced 10Cu/t-Zr, 10Cu/Al, and 10Cu/30Zr/Al samples (A) and Cu/30Zr/Al samples with different Cu contents (2.5–50 wt % Cu) (B).

The CO adsorption capacity and the positions and intensities of the IR bands in the 1800–1100 cm^–1^ range (Table S2) depended on the Cu content, as shown in Figure 4A,B. Adsorption of CO on the 10Cu/Al catalyst resulted in bands at 1657, 1432, and 1228 cm^–1^ (Figure 5A). The bands at 1657 and 1228 cm^–1^ were attributed to bidentate bicarbonates (b-HCO_3_^–^), formed due to the presence of OH groups on the surface,^46^ while the band at 1432 cm^–1^ was assigned to the formation of ionic carbonates (i-CO_3_^2–^).^36^ The 10Cu/30Zr/Al sample exhibited CO adsorption bands similar to those observed for 10Cu/Al, comparable to those for 10Cu/t-Zr (Figure 5A).

Therefore, for the *x*Cu/30Zr/Al series (Figure 5B), it could be inferred that for low Cu contents (2.5 and 5 wt % Cu), the bands at 1657, 1432, and 1228 cm^–1^ had the same assignments as those observed for the 10Cu/Al sample. However, for the catalysts with Cu contents above 5 wt % (Figure 5B), in addition to the 1657 and 1228 cm^–1^ bands (related to the presence of bidentate bicarbonates), there was a band at 1387 cm^–1^, attributed to the presence of carboxylate species (COOH).^36^ As shown in Figure 5B, the spectrum of the 50Cu/30Zr/Al sample exhibited low signal intensity, which could be explained by the dark color of the sample caused by the presence of a higher concentration of bulk CuO species.

**Figure 6:**
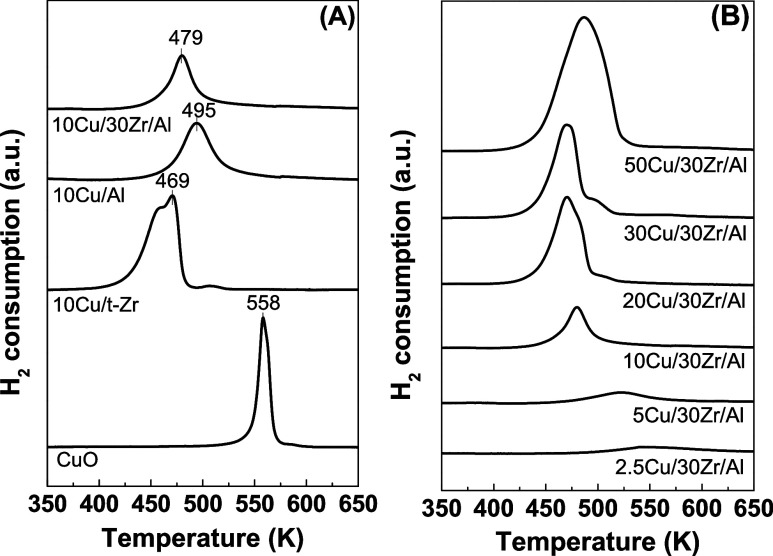
TPR profiles of the CuO, 10Cu/t-Zr, 10Cu/Al, and 10Cu/30Zr/Al samples (A) and Cu/30Zr/Al samples with different Cu contents (B).

Figure 6 shows the TPR profiles for CuO and the 10Cu/Al, 10Cu/t-Zr, and *x*Cu/30Zr/Al catalysts. For all of the catalysts, the reduction temperatures were lower than for CuO, which could be attributed to the interaction between the copper particles and the support. High dispersion resulted in a large surface area and, consequently, an increased number of defect sites where hydrogen activation could occur.^49^

The reduction profile of the 10Cu/t-Zr catalyst showed two distinct reduction regions. The reduction at a lower temperature (469 K) could be attributed to highly dispersed CuO on the support, with the interfacial reduction of CuO being facilitated by oxygen vacancy sites on the ZrO_2_ surface. The reduction at higher temperature (508 K) was associated with the presence of larger CuO particles. The reduction profiles of the 10Cu/Al and 10Cu/30Zr/Al catalysts exhibited a single peak, corresponding to the reduction of highly dispersed CuO on the support. However, for the 10Cu/30Zr/Al catalyst, the reduction occurred at a lower temperature (479 K), compared to the 10Cu/Al catalyst (495 K), suggesting a copper–zirconia interaction. ZrO_2_ facilitates copper reduction by the presence of anionic vacancies that activate gaseous H_2_ to adsorbed atomic hydrogen at the interface with copper. The activated hydrogen can then diffuse to the CuO surface, enabling its reduction.^26,50,51^ The lower hydrogen consumption for the 10Cu/30Zr/Al catalyst, compared to the 10Cu/Al catalyst (Table 3), could be explained by the higher efficiency of H_2_ activation at the zirconia vacancies.

**Table 3:** TPR Characteristics of CuO, 10Cu/t-Zr, 10Cu/Al, and Cu/30Zr/Al Samples with Different Cu Contents

samples	*T*_max_ (K)	H_2_ consumption (μmol/g_cat_)
2.5Cu/30Zr/Al	555	617.9
5Cu/30Zr/Al	523	675.0
10Cu/30Zr/Al	479	1068.9
20Cu/30Zr/Al	469	2326.1
30Cu/30Zr/Al	469	3512.3
50Cu/30Zr/Al	488	5204.1
10Cu/Al	495	1368.3
10Cu/t-Zr	469	2060.6
bulk CuO	558	5340.5

The reduction profiles for the *x*Cu/30Zr/Al catalysts showed distinct regions corresponding to different interactions between copper and the support. For the catalysts containing 2.5 and 5 wt % copper, the copper appeared to be highly dispersed as small particles or isolated ions that interacted strongly with the ZrO_2_/Al_2_O_3_ support. This strong interaction necessitated higher reduction temperatures, exceeding 523 K. At these lower copper contents, the reduction of CuO could involve the diffusion of H_2_ into the framework or the diffusion of oxygen from the framework to the surface.^26^ As the copper content increased to 30 wt %, the maximum reduction temperature (*T*_max_) shifted to lower values (Table 3), which could be attributed to the interaction between CuO and ZrO_2_, with CuO reduction facilitated by the presence of anionic vacancies on the zirconia surface.^26,35^

The TPR profiles for the catalysts with 20 and 30 wt % Cu contents showed the lowest reduction temperature (469 K) but also presented a shoulder in a higher-temperature region, at 498 K, corresponding to the reduction of larger CuO particles. This suggested that the catalyst featured a heterogeneous distribution of CuO species on the support, with the reduction of well-dispersed CuO species in moderate and strong interaction with the support. These findings were consistent with the XRD analyses (Figure 6B), revealing CuO segregation at copper contents exceeding 20 wt %. For the sample with the highest copper content (50 wt % Cu), there was a noticeable peak broadening and a shift of *T*_max_ to a higher temperature (488 K, Table 3). This shift could be explained by the increased crystallinity of CuO with higher copper contents, as evidenced by the XRD results.

The EXAFS parameters for the 10Cu/Al, 10Cu/t-Zr, and *x*Cu/30Zr/Al catalysts after reduction at 523 K under a flow of H_2_ are presented in Table 4 and Figures S2 and S3, including the average first nearest-neighbor coordination numbers for Cu–Cu (*N*_Cu–Cu_) and Cu–O (*N*_Cu–O_) and the Cu–Cu and Cu–O bond distances (*R*_Cu–Cu_ and *R*_Cu–O_). The copper particle size was calculated from the *N*_Cu–Cu_ and *R*_Cu–Cu_ values, assuming a cuboctahedral geometry.^52^ For fitting the EXAFS spectra, only the first coordination shell was considered.

**Table 4:** EXAFS Parameters for the Bulk Cu_2_O and Cu^0^ References and 10Cu/t-Zr, 10Cu/Al, and *x*Cu/30Zr/Al Catalysts Reduced at 523 K in a Flow of 5% H_2_/Ne

	references		samples					
	Cu_2_O	Cu^0^	5Cu/30Zr/Al	10Cu/30Zr/Al	20Cu/30Zr/Al	30Cu/30Zr/Al	10Cu/t-Zr	10Cu/Al
*E*_0_			2.3 (±0.2)	2.1 (±0.6)	1.9 (±0.1)	1.9 (±0.1)	2.5 (±0.2)	1.7 (±0.4)
*N*_Cu–Cu_		12	6.7 (±0.1)	7.1 (±0.2)	8.0 (±0.3)	8.4 (±0.1)	8.5 (±0.1)	6.7 (±0.2)
*N*_Cu–O_	2		1.1 (±0.1)	0.8 (±0.2)	0.7 (±0.2)	0.6 (±0.1)	0.6 (±0.2)	0.9 (±0.2)
*R*_Cu–Cu_ (Å)		2.54 (±0.002)	2.51 (±0.01)	2.50 (±0.01)	2.50 (±0.01)	2.51 (±0.01)	2.51 (±0.01)	2.50 (±0.01)
*R*_Cu–O_ (Å)	1.84 (±0.006)		1.84 (±0.01)	1.89 (±0.02)	1.94 (±0.01)	1.97 (±0.02)	1.99 (±0.02)	1.89 (±0.01)
σ_Cu–Cu_^2^ (Å^2^)		0.009 (±0.0002)	0.0145 (±0.0002)	0.0147 (±0.0006)	0.0148 (±0.0006)	0.0149 (±0.0001)	0.0144 (±0.0001)	0.0147 (±0.0005)
σ_Cu–O_^2^ (Å^2^)	0.002 (±0.0008)		0.009 (±0.001)	0.008 (±0.005)	0.012 (±0.006)	0.013 (±0.003)	0.014 (±0.004)	0.008 (±0.004)
a*d*_p_ (nm)			3.6	4.0	5.0	5.6	3.7	3.5
%Cu^0^			70	75	85	95	95	72

a *d*_p_: particle size using the cuboctahedral model.

The Cu–Cu bond distances for the samples were approximately 2.50 Å, smaller than *R*_Cu–Cu_ for metallic copper (2.54 Å). The electronic structures of the atoms in metal nanoparticles differ from those in the bulk metal because spd rehybridization increases the electron density between the metal atoms, leading to a contraction of the metal–metal bond distances.^53^

The *N*_Cu–Cu_ values of the samples ranged from 6.7 to 8.5, which could be compared to a value of 12 for metallic copper foil (Table 4). The ratio of surface atoms to bulk atoms is higher for nanoparticles compared to Cu^0^ foil. Hence, the lower values for the samples reflected that the copper species were highly dispersed on the support, so the surface atoms were not bonded to the maximum number of nearest neighbors, resulting in fewer coordinated atoms.

The *N*_Cu–Cu_ values for the 5Cu/30Zr/Al and 30Cu/30Zr/Al samples were 6.7 and 8.5, respectively (Table 4), showing that the increase in the copper content led to a higher Cu–Cu coordination number. Consequently, increasing the copper content from 5 to 30 wt % resulted in the copper particle size increasing from 3.6 to 5.6 nm. All of the samples presented a contribution from Cu–O scattering, suggesting that they were slightly oxidized after the reduction process. To quantify the degree of copper reduction, linear combination fitting of the XANES spectra for the samples was performed with reference standards (Cu^0^, Cu_2_O, and CuO). The results for all of the samples (Table 4) indicated that they were not completely reduced, with the degree of reduction ranging from 70% for 5Cu/30Zr/Al to 95% for 30Cu/30Zr/Al. Therefore, the amount of metallic copper increased as the copper content increased, indicating that larger copper particles tended to be more reduced. This trend was evident in the EXAFS parameters (Table 4), where the *N*_Cu–Cu_/*N*_Cu–O_ ratio increased with copper content due to the decreasing oxygen contribution, further supporting the findings from the linear combination fitting. These observations were in agreement with the work of Knapp et al.,^36^ who reported that the degree of reduction varied systematically with copper particle size. In addition, the Cu–O bond distance increased from 1.84 Å for 5Cu/30Zr/Al to 1.97 Å for 30Cu/30Zr/Al, indicating that the bonding of oxygen was weaker in larger particles.

The EXAFS spectra fitting showed no Cu–Cu scattering corresponding to the Cu_2_O structure. Furthermore, the similarity among the samples for the Cu–Cu bond distance, regardless of the degree of reduction, suggested that the Cu nanoparticles might be composed of a reduced core with some oxygen on the surface. The surface Cu^+^/Cu^0^ ratio appeared to correlate with particle size since this ratio was smaller for larger particles.

### 3.2 Catalytic Performance of the *x*Cu/30Zr/Al Catalysts in the Ethanol Conversion Reaction and Relation to Physicochemical Properties

The effects of reaction temperature and copper content on ethanol conversion and product selectivity are summarized in Table 5. The main products were ethyl acetate (EtOAc) and acetaldehyde (AcH), with coproducts including ethylene (ETE), diethyl ether (DEE), methyl ethyl ketone (MEK), crotonaldehyde (CROT), and minor amounts of CO and CO_2_. For all of the samples, ethanol conversion increased with temperature. For example, the 5Cu/30Zr/Al catalyst showed 48.0% conversion at 473 K, with an increase to 89.4% at 548 K. For all of the temperatures tested, superior conversion performance was observed for the catalysts with higher copper loadings (>5 wt % Cu).

**Table 5:** Effect of Cu Content on the Behavior of the *x*Cu/30Zr/Al Catalysts in Ethanol Conversion at Different Reaction Temperatures

			selectivity (%)								
temperature (K)	catalysts	*X*_EtOH_ (%)	CO	CO_2_	ETE	AcH	PROP	DEE	MEK	AcOEt	CROT
473	2.5Cu/30Zr/Al	35.8	0.3	0.6	n.d.	41.9	n.d.	14.1	7.9	23.5	11.7
	5Cu/30Zr/Al	48.0	0.5	0.3	n.d.	24.7	0.4	16.5	7.9	39.9	9.8
	10Cu/30Zr/Al	50.1	0.6	0.2	n.d.	20.6	0.3	15.0	4.8	52.7	5.7
	20Cu/30Zr/Al	50.7	0.3	0.3	n.d.	19.3	0.4	13.3	4.7	56.6	4.7
	30Cu/30Zr/Al	51.1	0.6	0.3	0.3	16.5	0.5	12.0	4.0	62.8	3.1
	50Cu/30Zr/Al	51.2	0.4	0.2	0.4	23.6	0.4	10.2	5.2	55.0	4.4
	10Cu/Al	46.5	0.3	0.2	0.2	28.4	--	15.4	8.5	34.0	12.8
	10Cu/t-Zr	41.7	0.4	0.6	0.3	36.2	0.6	n.d.	12.0	42.5	7.4
498	2.5Cu/30Zr/Al	58.4	0.2	0.5	0.9	38.5	0.6	15.9	9.7	25.5	8.3
	5Cu/30Zr/Al	64.0	0.4	0.5	0.3	24.2	0.7	16.6	7.8	41.7	7.9
	10Cu/30Zr/Al	66.2	0.7	0.5	0.4	18.5	0.9	13.6	5.5	56.0	4.0
	20Cu/30Zr/Al	67.5	0.6	0.5	0.5	17.1	1.0	10.8	4.6	61.8	3.1
	30Cu/30Zr/Al	69.3	0.5	0.6	0.8	14.6	1.2	8.2	4.1	67.8	2.1
	50Cu/30Zr/Al	69.1	0.7	0.5	0.4	21.6	0.9	10.1	5.4	57.3	3.2
	10Cu/Al	68.1	0.7	0.7	0.4	26.1	1.0	14.0	9.9	39.1	8.2
	10Cu/t-Zr	63.8	0.8	1.2	1.2	31.0	1.3	n.d.	12.0	46.7	5.8
523	2.5Cu/30Zr/Al	76.2	0.3	2.0	0.6	37.1	1.4	15.8	10.7	26.3	5.9
	5Cu/30Zr/Al	80.2	0.7	1.3	0.5	23.2	2.0	15.2	6.4	45.8	4.9
	10Cu/30Zr/Al	80.3	0.8	1.2	0.6	17.6	2.2	11.6	5.1	58.0	2.8
	20Cu/30Zr/Al	81.8	0.7	1.2	0.7	16.0	2.3	8.7	4.6	63.9	1.9
	30Cu/30Zr/Al	81.9	1.2	1.6	0.7	14.1	2.9	6.9	4.1	67.2	1.5
	50Cu/30Zr/Al	81.8	1.0	1.1	0.6	21.1	2.1	8.5	5.3	58.3	2.1
	10Cu/Al	80.2	1.1	1.6	0.7	24.9	2.6	12.8	9.1	42.1	5.1
	10Cu/t-Zr	77.9	0.9	2.2	0.6	28.5	3.0	n.d.	11.2	50.1	3.6
548	2.5Cu/30Zr/Al	86.8	0.4	1.0	4.5	36.0	3.4	14.3	10.6	26.5	3.2
	5Cu/30Zr/Al	89.4	1.2	3.4	4.5	20.7	5.3	13.4	8.1	40.5	2.9
	10Cu/30Zr/Al	90.9	1.4	3.2	1.3	17.9	7.4	10.3	6.5	50.2	1.8
	20Cu/30Zr/Al	90.9	1.4	4.0	1.2	17.2	7.8	8.2	6.0	52.9	1.4
	30Cu/30Zr/Al	91.0	1.5	5.0	1.2	16.4	8.0	5.7	5.8	55.1	1.4
	50Cu/30Zr/Al	89.6	1.1	3.1	1.1	20.5	6.0	8.1	6.0	52.7	1.4
	10Cu/Al	89.7	1.2	4.5	1.3	21.8	7.2	10.5	8.8	39.1	5.6
	10Cu/t-Zr	86.9	1.4	4.2	0.9	27.0	5.7	n.d.	11.1	47.8	2.0

**Figure 7:**
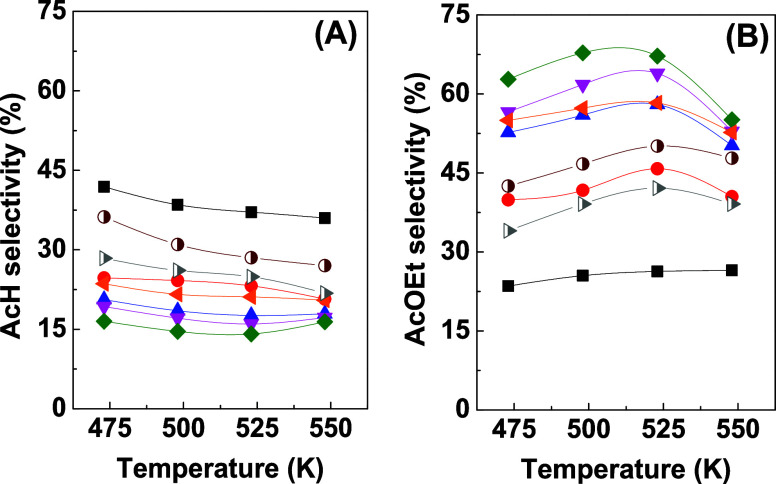
Selectivity toward acetaldehyde (A) and ethyl acetate (B) for 10Cu/Al (half-filled right-facing gray triangle), 10Cu/t-Zr (half-filled maroon circle), and *x*Cu/30Zr/Al catalysts with 2.5 wt % Cu (black square solid), 5 wt % Cu (red circle solid), 10 wt % Cu (blue triangle solid), 20 wt % Cu (pink triangle down solid), 30 wt % Cu (green tilted square solid), and 50 wt % Cu (solid left-facing orange triangle) as a function of temperature.

Figure 7 shows the selectivity toward acetaldehyde and ethyl acetate as a function of the reaction temperature. The ethyl acetate selectivity increased as the copper content of the *x*Cu/30Zr/Al catalysts increased up to 30 wt % Cu, while the catalyst with the highest copper content, 50Cu/30Zr/Al, exhibited lower selectivity than the 30Cu/30Zr/Al sample. A comparison of the ethyl acetate selectivity of the catalysts with 10 wt % copper on different supports at 523 K (Figure 7A) showed that the 10Cu/30Zr/Al catalyst presented the highest selectivity of ∼58%, compared to 42.1% for 10Cu/Al and 50.1% for 10Cu/t-Zr. For all of the catalysts, the ethyl acetate selectivity increased in the temperature range from 473 to 523 K, followed by a decrease in selectivity at 548 K (Figure 7B).

High acetaldehyde selectivity was observed for the *x*Cu/30Zr/Al catalysts with low copper contents (2.5 and 5 wt % Cu) (Figure 7A), followed by the 50Cu/30Zr/Al catalyst with the highest copper content. Comparing the different supports with the same copper content of 10 wt % Cu, the order of selectivity at all reaction temperatures was 10Cu/t-Zr > 10Cu/Al > 10Cu/30Zr/Al. The lowest acetaldehyde selectivity was shown by the 30Cu/30Zr/Al catalyst.

**Figure 8:**
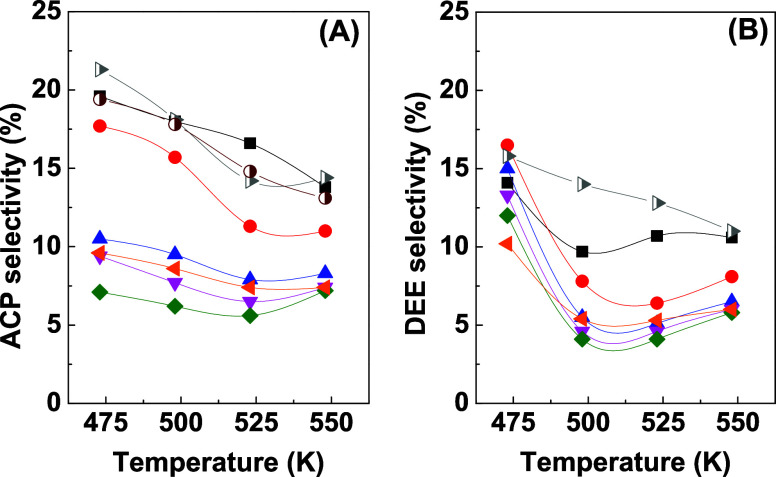
Selectivity toward aldol condensation products (A) and diethyl ether (B) for 10Cu/Al (half-filled right-facing gray triangle), 10Cu/t-Zr (half-filled maroon circle), and *x*Cu/30Zr/Al catalysts with 2.5 wt % Cu (black square solid), 5 wt % Cu (red circle solid), 10 wt % Cu (blue triangle solid), 20 wt % Cu (pink triangle down solid), 30 wt % Cu (green tilted square solid), and 50 wt % Cu (solid left-facing orange triangle) as a function of temperature.

Selectivity toward the aldol condensation products (ACPs) (Figure 8), methyl ethyl ketone and crotonaldehyde, was higher for the *x*Cu/30Zr/Al catalysts with low copper contents (2.5 to 5 wt % Cu) and for the 10Cu/Al and 10CuAl/t-Zr catalysts.

The 10Cu/Al catalyst exhibited the highest selectivity toward diethyl ether (Figure 8B). In contrast, no diethyl ether formation was observed on the surface of the 10Cu/t-Zr catalyst (Table 5). Among the catalysts in the *x*Cu/30Zr/Al series, the highest diethyl ether selectivity was obtained for the samples with the lowest copper contents (2.5 and 5 wt % Cu).

**Figure 9:**
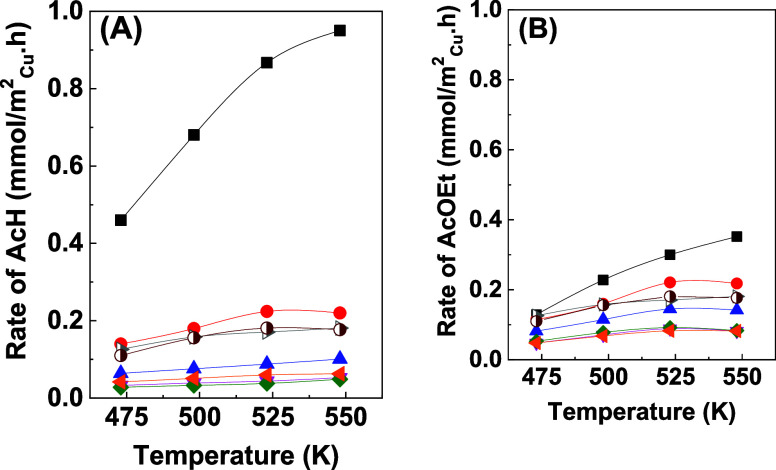
Rates of formation of acetaldehyde (A) and ethyl acetate (B) for 10Cu/Al (half-filled right-facing gray triangle), 10Cu/t-Zr (half-filled maroon circle), and *x*Cu/30Zr/Al catalysts with 2.5 wt % Cu (black square solid), 5 wt % Cu (red circle solid), 10 wt % Cu (blue triangle solid), 20 wt % Cu (pink triangle down solid), 30 wt % Cu (green tilted square solid), and 50 wt % Cu (solid left-facing orange triangle) as a function of temperature.

Figure 9 shows the effects of copper loading and reaction temperature on the rates of formation of acetaldehyde and ethyl acetate using the different catalysts. Superior performance was observed for the catalyst with 2.5 wt % Cu, which provided the highest rates for both products. The acetaldehyde formation rate increased sharply with temperature, reaching nearly 1.0 mmol/m_Cu_^2^·h at 550 K, while the ethyl acetate formation rate showed a more gradual increase, with a maximum of approximately 0.4 mmol/m_Cu_^2^·h. The catalysts with higher copper loadings showed significantly lower activities, suggesting an optimal copper content, above which particle agglomeration or surface saturation could have occurred, reducing the number of active sites.

**Figure 10:**
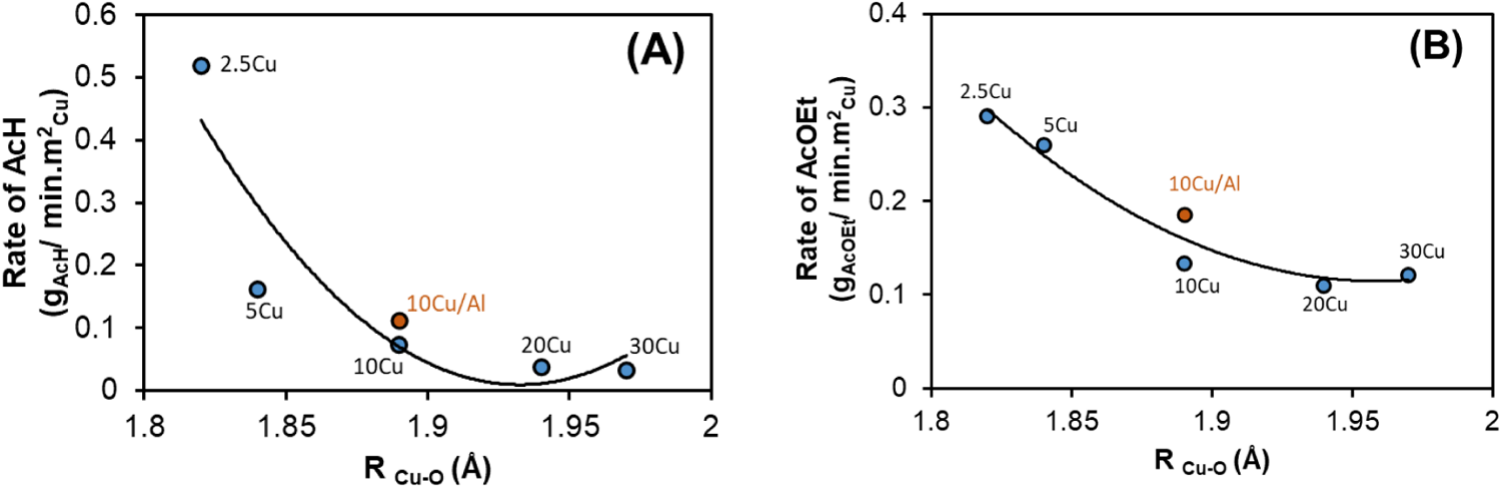
Relation between the Cu–O bond distance (*R*_Cu–O_) and the catalytic activity of the *x*Cu/30Zr/Al and 10Cu/Al catalysts. Rate of acetaldehyde (AcH) formation (g_AcH_/min·m_Cu_^2^) as a function of *R*_Cu–O_ (A). Rate of ethyl acetate (AcOEt) formation (g_AcOEt_/min·m_Cu_^2^) as a function of *R*_Cu–O_ (B). The catalytic tests were conducted at 473 K. The Cu–O bond distance for the 2.5Cu sample was extrapolated.

Figure 10 shows the relationships between the Cu–O bond distance (*R*_Cu–O_) and the rates of formation of acetaldehyde (AcH) and ethyl acetate (AcOEt). This analysis highlighted the effects of Cu^0^ particle size and surface electronic properties on catalytic activity. The effect of particle size involved two opposing mechanisms influencing the surface electron density: (i) the smaller particle size was associated with increased electron density due to quantum confinement effects and (ii) the higher density of Cu^+^–O species on the surface, resulting from increased reactivity with oxygen, decreased the electron density on the Cu^0^ surface.^20,54^ These effects together determined the final electronic properties of Cu^0^. The Cu–O bond distance served as a probe for the surface electron density of Cu^0^, with shorter distances indicating a predominant transfer of electron density from Cu^0^ to Cu^+^–O species.

## 4 Discussion

The XPS and DRIFTS-CO analyses showed that the catalysts with low copper contents (2.5 and 5 wt % Cu) exhibited a predominance of Cu^+^ species over Cu^0^ species on the catalyst surface. The prevalence of Cu^+^ species could be attributed to the increased dispersion of the active phase as the copper content decreased, which was probably due to the high surface area provided by the support. In addition, these catalysts presented lower metallic copper surface areas, as confirmed by N_2_O titration (Table 1).

The results for the 2.5Cu/30Zr/Al and 5Cu/30Zr/Al catalysts indicated that increased copper dispersion led to higher Cu binding energy and lower values of the Auger parameter (α_Cu_). These changes suggested a high concentration of Cu^+^ species on the catalyst surface. The shift of the valence band to higher binding energy values could be attributed to the reduced particle size.^55^ As confirmed by EXAFS (Table 4), these catalysts exhibited the smallest metallic particle sizes. Isolated Cu atoms or small particles at the metal–support interface can acquire a partially positive charge due to electron transfer from the metal to the support.^55^

Furthermore, according to the DRIFTS-CO analyses (Figures 3 and 4), the presence of the band at 2011 cm^–1^ could be attributed to Cu^+^ sites with low electron density. Higher intensity of this band was observed for the catalysts containing 2.5 and 5 wt % of supported copper, compared to other catalysts in the same series.

The DRIFTS-CO results, together with those for N_2_O titration, revealed that the concentration of Cu^+^ species was higher for the 2.5Cu/30Zr/Al catalyst, compared to 5Cu/30Zr/Al, as evidenced by the higher intensity of the band at 2011 cm^–1^. Lower dispersion (68%) observed for the 2.5Cu/30Zr/Al catalyst indicated that the Cu^+^ species were stabilized on the support after reduction. The findings from XPS and DRIFTS-CO were consistent with those from EXAFS, showing a decrease in the degree of reduction with decreasing Cu content, as indicated by higher Cu–O coordination numbers.

For supported Cu contents above 5 wt % on 30Zr/Al, the XPS data revealed the presence of Cu^+^ and Cu^0^ species on the surface support, with the Cu^+^/Cu^0^ ratio varying according to the copper content (Figure 2B). An equilibrium between Cu^+^ and Cu^0^ species was reached at around 20 to 30 wt % of supported Cu. The X-ray diffractograms indicated that although the catalysts retained a high surface area, a heterogeneous distribution of particle sizes was likely for copper contents above 10 wt % due to the onset of CuO agglomeration. For catalysts with Cu contents of 20 and 30 wt %, lower reduction temperatures were observed, suggesting an interaction between CuO and the ZrO_2_ support. This could be attributed to the facilitated reduction of interfacial Cu species within anionic vacancies of the ZrO_2_ support. An increase in the degree of reduction for these samples was confirmed by EXAFS, which showed a reduced coordination number, N_Cu–O_, accompanied by increases of *N*_Cu–Cu_ and *r*_Cu–O_ with increasing copper content (Table 4). However, a fraction of the Cu^+^ species remained on the catalyst surface. These findings were consistent with the proposed hypothesis that the electron density of superficial Cu atoms would increase with higher copper content, with metallic Cu particles being predominant on the surfaces of the catalysts with high Cu loadings.

The composition of Cu^0^ and Cu^+^ species on the catalyst surface may result from a dispersive effect, as observed by Knapp et al.,^36^ who suggested that the formation of oxidized species could occur not only at the metal–support interface but also on the surfaces of metallic copper nanoparticles, with the oxygen concentration being influenced by particle size and dispersion. Similarly to observations for the Cu/TiO_2_ catalyst,^56^ this effect can result in oxygen transfer from the ZrO_2_ support to the surfaces of the copper particles due to the oxygen-rich interface generated by the spillover mechanism between copper and zirconia during the reduction process.

The results of the catalytic tests indicated that the product distribution during ethanol conversion was strongly influenced by the electronic properties of the copper particles and the acidity/basicity of the support surface. The high formation of acetaldehyde observed for the catalysts with low copper contents (Table 5) could be explained by the high density of Cu^+^ sites on the surfaces of these catalysts. According to the literature,^17^ the activity of Cu^+^ species in the dehydrogenation of ethanol can be attributed to the adsorption of ethanol to form ethoxide species (CH_3_CH_2_O–Cu^+^). After losing hydrogen due to abstraction by a neighboring Cu^+^ atom, these species desorb from the surface as acetaldehyde. Therefore, for acetaldehyde formation to occur, nearby Cu^+^ sites are required. Hence, the high concentrations of Cu^+^ species on the surfaces of the 2.5Cu/30Zr/Al and 5Cu/30Zr/Al catalysts allowed the formation of nearby Cu^+^ sites highly selective for acetaldehyde production. The prevalence of metallic copper on the surfaces of the catalysts with higher Cu contents favored the adsorption of acyl species (CH_3_CO–Cu^0^) formed during the dissociative adsorption and dehydrogenation of acetaldehyde on Cu^0^ sites.^57^

For pure ZrO_2_, DFT studies indicate a high energy barrier for breaking the ^β^C–H bond of ethanol.^58^ As a result, ethoxide species (CH_3_CH_2_O–Zr^δ+^) were adsorbed on Zr^δ+^ sites on the support surface, as confirmed by XPS analysis. The support also played an important role in the formation of ethanol conversion products due to the presence of acidic and basic sites. Ethanol dehydration reactions on the surface of alumina led to the formation of diethyl ether and ethylene (Table 5), while the acid sites of alumina also favored the formation of crotonaldehyde.

Acetaldehyde intermediates formed on copper particles could be transferred to the ZrO_2_ surface, primarily by diffusion (spillover), due to the creation of an interface that facilitated the formation of condensation products from acetaldehyde.

An indication that the deposition of ZrO_2_ over Al_2_O_3_ did not completely cover the alumina surface was the observation (shown in Figure 7) that the selectivity toward diethyl ether did not drastically decrease, which would have been expected with the addition of approximately 30 wt % ZrO_2_ over alumina. According to Damyanova et al.,^27^ the formation of a monolayer on alumina requires about 17 wt % of ZrO_2_. The XRD data (Figure 1B) showing a pronounced peak for tetragonal ZrO_2_ suggested that the ZrO_2_ did not fully cover the support and instead formed large crystallites on the alumina surface.

For the *x*Cu/30Zr/Al catalysts, the selectivity toward ethyl acetate resulted from the combined contributions of copper sites on the surfaces of both Al_2_O_3_ and ZrO_2_. The DRIFTS-CO analyses (Figures 3 and 4) enabled the identification of distinct copper sites on alumina and zirconia. Previous work reported that catalysts with copper particles supported on monoclinic zirconia presented sites highly selective for ethyl acetate, compared to catalysts with copper supported on tetragonal or amorphous zirconia, due to the superior redox properties of the monoclinic phase.^18^ However, work by Ram and Mondal^59^ indicated that the Al^3+^ ions present in alumina can influence the formation of zirconia, directing it toward the tetragonal phase.

Aldol condensation reactions can occur on the surface of ZrO_2_, where the condensation of acetaldehyde (β-aldolization) is facilitated by the presence of basic sites that abstract α-hydrogen, together with strong Lewis acid sites that enable the condensation of two acetaldehyde molecules.^60^

**Figure 11:**
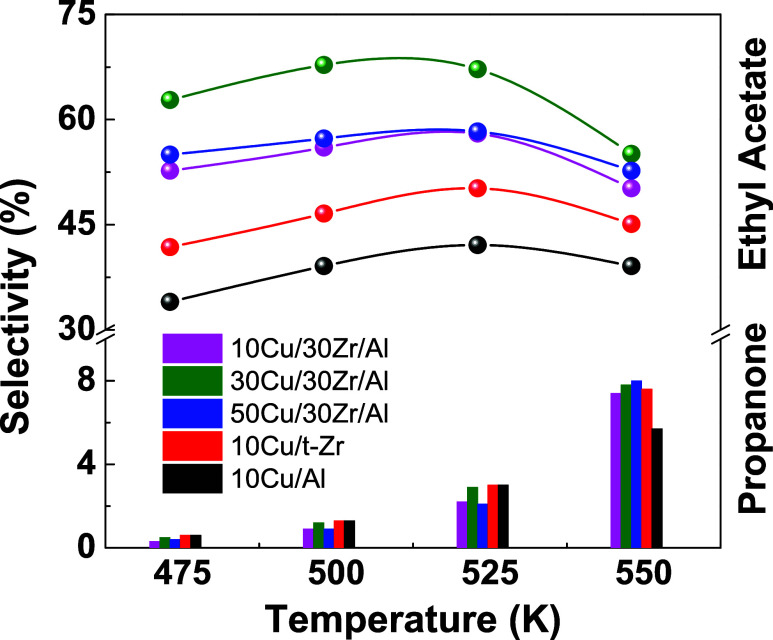
Evolution of the selectivity to propanone and ethyl acetate as a function of the reaction temperature for the 10Cu/Al, 10Cu/t-Zr, and *x*Cu/30Zr/Al catalysts.

At higher temperatures (above 523 K), all of the catalysts showed a loss of specificity toward ethyl acetate, correlating with the formation of propanone (Figure 11). This behavior could be attributed to subsequent oxidation pathways involving acetaldol, an intermediate formed during aldol condensation. As reported by Pennella and Elliott^61^ for Cu/ZnO/Al_2_O_3_ catalysts, acetaldol can undergo oxidation on the catalyst surface, forming a carboxylate species by interaction with adsorbed oxygen (O_s_). This carboxylate intermediate then decomposes into propanone, CO_2_, and H_2_, as shown in the following reaction pathway
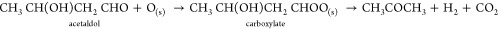


The coupling of surface hydroxyl groups (2OH_(s)_^–^ → O_(s)_ + H_2_O) by proton transfer may occur, leading to the formation of atomic oxygen within the lattice. This reaction was consistent with the observed changes in product distributions with the increase of temperature using the *x*Cu/30Zr/Al catalysts. At lower temperatures, the formation of ethanol dehydration products, such as diethyl ether and water, takes place. As the reaction temperature increased, the selectivity toward diethyl ether declined (Figure 8), suggesting that the dehydration reaction became less dominant.

The rapid consumption of oxygen during the formation of ethyl acetate and propanone could deplete oxygen from ZrO_2_, inducing changes in the electron density of copper particles that affected the catalytic activity and could explain the observed decrease in selectivity toward ethyl acetate. Notably, the ketonization reaction could compete with the ethanol and acetaldehyde condensation pathway for the surface-adsorbed oxygen, which is essential for transporting ethoxide species required in ethyl acetate synthesis. Simultaneously, the atomic oxygen generated during these processes could facilitate secondary oxidation reactions, further favoring the formation of propanone at the expense of ethyl acetate selectivity.

Above 523 K, due to a shift in the reaction dynamics and a sharp decrease in the selectivity of the *x*Cu/30Zr/Al catalysts toward ethyl acetate, another mechanism might be involved in replenishing the adsorbed oxygen on the surface.^18^ Surface oxygen could be formed by the reaction between a hydroxyl group adsorbed on a Zr^4+^ cation and a hydrogen anion adsorbed on Zr^3+^, as follows: OH^–^ + H^–^ → H_2(g)_ + O^2–^. Rapid consumption of the surface oxygen in the oxidation of acetaldol would then lead to increased selectivity toward acetone.

The creation of a metal–oxide interface is a critical factor for ethyl acetate formation and for the promotion of aldol condensation. Several studies concerning ethanol conversion have suggested that the direct transformation of ethanol to ethyl acetate may occur by the condensation of an ethoxide species (CH_3_CH_2_O*), preferentially adsorbed on Cu^+^ and Zr^δ+^ sites, and an acyl species (CH_3_CO*), predominantly adsorbed on Cu^0^ sites.^17−19^ Although the proximity of Cu^0^ and Cu^+^ sites could enhance ethyl acetate formation on the copper particles, the most selective sites for ethyl acetate production were probably the Cu^0^–Zr^δ+^ sites. As shown in Table 5, the 20Cu/30Zr/Al and 30Cu/30Zr/Al catalysts presented the highest selectivity toward ethyl acetate.

The increase in ethyl acetate selectivity as a function of the copper content suggested that the formation of sites highly selective toward ethyl acetate required an optimal Cu^+^/Cu^0^ ratio and the presence of basic sites. At lower copper loadings (2.5 and 5 wt % Cu), there was increased formation of acetaldehyde and crotonaldehyde, together with lower ethyl acetate production (Table 5). This was accompanied by greater formation of ethanol dehydration products, probably due to the increased exposure of alumina, which is more active for dehydration reactions.

The increase of copper loading favored ethyl acetate formation, while the production of acetaldehyde and crotonaldehyde decreased, suggesting that the reaction mainly occurred at the metal–oxide interface. If the reaction occurred predominantly on the support, greater exposure of the support would result in higher ethyl acetate formation, as observed for crotonaldehyde. Remarkably, ethyl acetate formation exhibited an inverse trend to crotonaldehyde formation, with the highest selectivity for an optimal copper loading of 30 wt %. However, at higher copper loadings (for the 50Cu/30Zr/Al catalyst), complete coverage of the support led to the blocking of the interface sites, reducing selectivity for ethyl acetate. The increase in copper particle size, as indicated by the XRD, TPR, EXAFS, and XPS analyses, enhanced the formation of the metal–oxide interface while suppressing aldol condensation byproduct formation on the support.

For the *x*Cu/30Zr/Al catalysts, the rate of ethyl acetate formation exceeded that of acetaldehyde (Figure 9), indicating that acetaldehyde was rapidly consumed in ethyl acetate formation. However, for low copper loadings on the 30Zr/Al support, acetaldehyde formation rates were higher, creating a surface rich in ethoxide species. Due to a deficiency of acyl species, condensation between acyl and ethoxide species was less efficient, resulting in a reduced rate of ethyl acetate formation. Ethoxide species adsorbed on the surface were likely to be transferred to the support by diffusion, leading to the formation of byproducts.

As shown in Figure 10, lower *R*_Cu–O_ for catalysts with smaller Cu^0^ particles was associated with a decrease of surface electron density, reflecting the dominance of Cu^0^–O interactions over quantum confinement effects. In Cu/ZrO_2_/Al_2_O_3_ catalysts, a high density of ZrO–OCu^+^–Cu^0^ species at the interface is essential for catalytic activity. This interface facilitates ethanol activation, forming ethoxide intermediates that drive subsequent dehydrogenation and condensation reactions. However, a high density of these species also contributes to reduced electron density on the Cu^0^ surface, potentially lowering catalytic activity.

A comparison of Cu/SiO_2_^20^ and Cu/ZrO_2_/Al_2_O_3_ catalysts provides further evidence that for the Cu/ZrO_2_/Al_2_O_3_ system, the higher density of ZrO–OCu^+^–Cu^0^ species is crucial for achieving high catalytic activity in ethanol dehydrogenation.^62^ Unlike Cu/SiO_2_, where activity increases with Cu^0^ particle size, in the case of Cu/ZrO_2_/Al_2_O_3_ catalysts, control of the ZrO_2_–Cu^0^ interface is a key determinant of catalytic performance. Therefore, balancing the surface electron density of Cu^0^ with the density of ZrO–OCu^+^–Cu^0^ species at the interface is essential for optimizing ethanol conversion to value-added products.

## 5 Conclusions

Cu-based catalysts supported on ZrO_2_–Al_2_O_3_ showed significant activity for the selective conversion of ethanol to ethyl acetate. However, the selectivity toward ethyl acetate was strongly dependent on the copper content. Variations in Cu loading resulted in a heterogeneous distribution of particle sizes, which influenced the metal–oxide interface and the electron density of Cu particles (Cu^+^/Cu^0^ species), as characterized by DRIFTS-CO and XPS analyses.

A higher Cu^+^/Cu^0^ ratio, observed with a lower catalyst Cu content, favored the selective formation of acetaldehyde, the primary product of ethanol dehydrogenation. In contrast, higher Cu loadings led to a predominance of Cu^0^ species on the catalyst surface, resulting in greater coverage of the support, reduced byproduct formation from aldol condensation, and enhanced selectivity toward ethyl acetate. This improvement was attributed to the increased presence of acyl species at the metal–oxide interface. The ZrO–OCu^+^–Cu^0^ interface enhanced ethanol activation and ethoxide formation, promoting dehydrogenation and condensation, but excessive species density reduced the Cu^0^ electron density, potentially lowering activity. These findings highlight that the Cu/ZrO_2_–Al_2_O_3_ catalyst system can be optimized by adjusting the copper content and ensuring metal–oxide interactions, enabling efficient ethyl acetate production with minimal byproducts and offering a sustainable solution for ethanol valorization in industry.

## Supporting Information Available

Deconvoluted Cu LMM Auger spectra and Cu^0^/Cu^+^ quantification (Figure S1 and Table S1), EXAFS fits for Cu standards and catalysts (Figures S2 and S3), and DRIFTS-CO band assignments and references (Table S2) (PDF)

## Abbreviations

X_EtOH_: ethanol conversion
AcH: acetaldehyde
EtOAc: ethyl acetate
MEK: methyl ethyl ketone and/or butanol
DEE: diethyl ether
PROP: propanone
ETE: ethylene
CROT: crotonaldehyde
n.d.: not detected
