# Supplementary material for: Tailoring Cu-Based Catalysts Supported on ZrO2–Al2O3 for Efficient and Selective Ethanol Conversion to Ethyl Acetate
Source: ACS Mater Au. 2025 Apr 10;5(3):593–608. doi: 10.1021/acsmaterialsau.5c00017 (PMC12082363; doi:10.1021/acsmaterialsau.5c00017)
Supplement: Supplementary file 1 — mg5c00017_si_001.pdf [file mg5c00017_si_001.pdf]

-Supplementary Material-

Tailoring Cu-based catalysts supported on ZrO_2-Al_2O_3 for efficient and selective ethanol conversion to ethyl acetate

Isabel C. Freitas[a], Davi D. Petrolini[b], Jean Marcel R. Gallo[a], Paula C. P. Caldas[b], Daniela C. de Oliveira[c], João B. O. Santos[b], Clelia Mara de Paula Marques[a], José Maria Correa Bueno[b]*

[a]*Departamento de Química, Universidade Federal de São Carlos, C.P. 676, 13565-905 São Carlos, São Paulo, Brazil*

[b]*Departamento de Engenharia Química, Universidade Federal de São Carlos, C.P. 676, 13565-905 São Carlos, São Paulo, Brazil*

[c]*Laboratório Nacional de Luz Síncrotron, C.P. 6192, 13083-970 Campinas, São Paulo, Brazil*

*Corresponding author.

E-mail: jmcb@ufscar.br

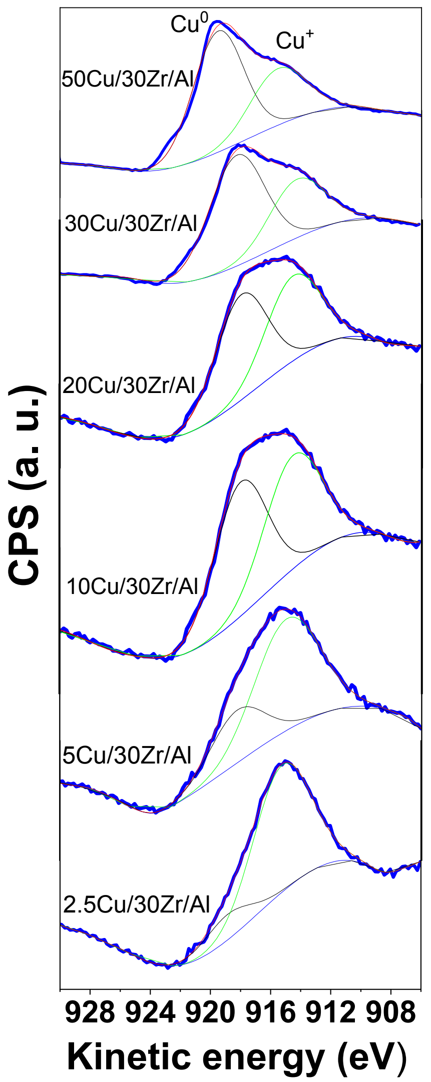

Figure S1. Deconvoluted Cu LMM Auger spectra of the reduced xCu/30Zr/Al catalysts.

Table S1. Surface Cu^0 and Cu^+ species for reduced xCu/30Zr/Al catalysts, estimated from Cu LMM Auger peak deconvolution (Figure S1).

	**Cu^0 area (a.u.)**	**Cu^+ area (a.u.)**	**Cu^0 (%)**	**Cu^+ (%)**
**50 Cu/30Zr/Al**	802424	414258	66	34
**30Cu/30Zr/Al**	396172	208779	65	35
**20Cu/30Zr/Al**	201659	172103	54	46
**10Cu/30Zr/Al**	209550	192612	52	48

**5Cu/30Zr/Al**	113961	288843	28	72
**2.5Cu/30Zr/Al**	32339	195721	14	86

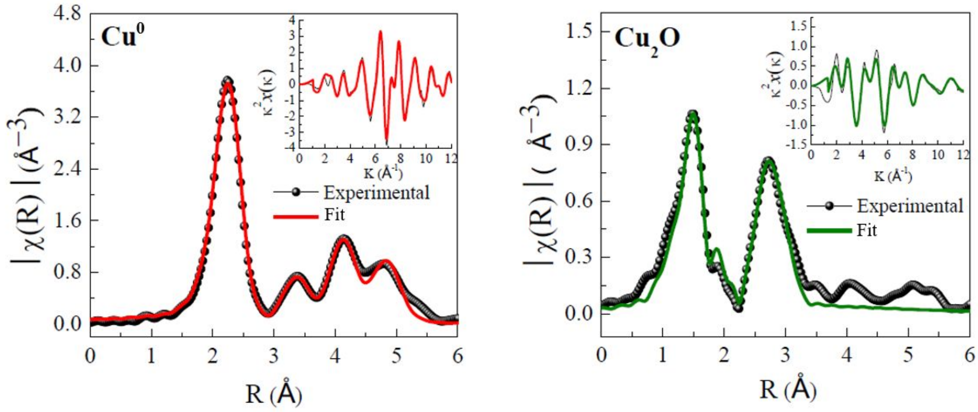

Figure S2. EXAFS oscillations at the Cu K-edge, obtained experimentally, and the fits performed in R-space and K-space (insets) for the Cu^0 and Cu_2O standards.

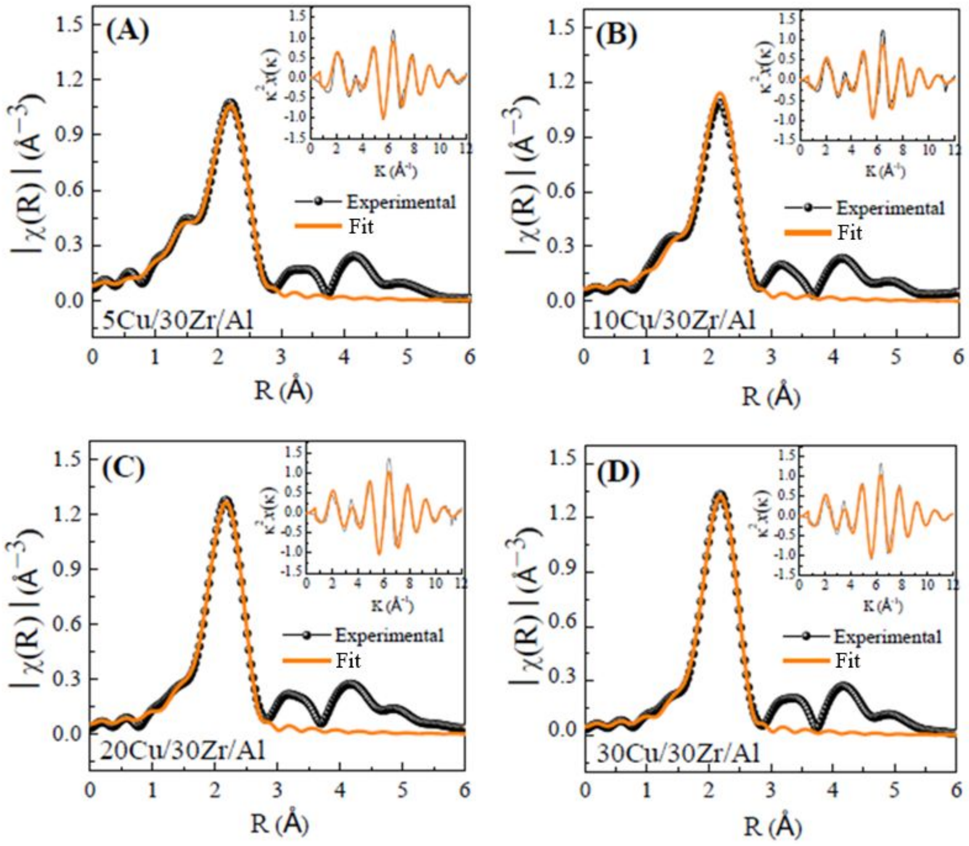

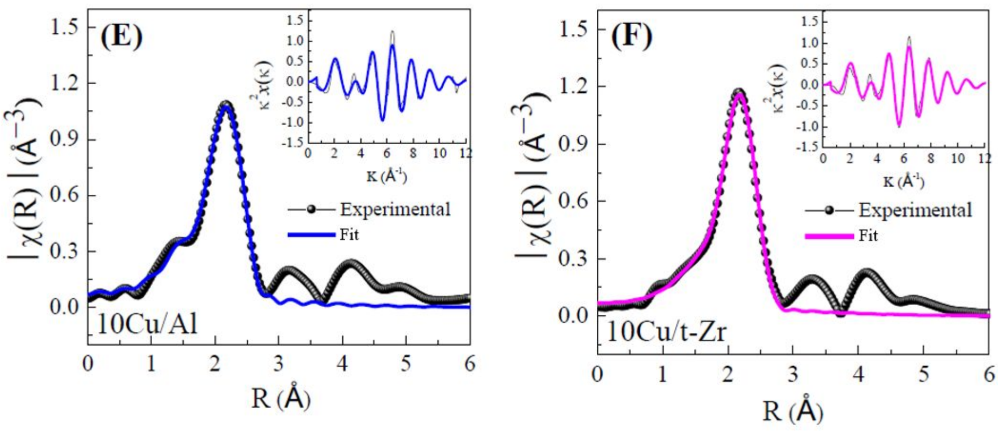

Figure S3. EXAFS oscillations at the Cu K-edge, obtained experimentally, and the fits performed in R-space and K-space (insets) for the *x*Cu/30Zr/Al, 10Cu/Al, and 10Cu/t-Zr catalysts.

Table S2. Positions of the DRIFTS bands of adsorbed CO for the *x*Cu/30Zr/Al, 10Cu/Al, and 10Cu/t-Zr catalysts.

Adsorbed species		Wavenumber (cm^{-1})	References
b-HCO_3^-<br>Bidentate bicarbonate	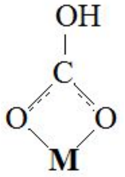	1657, 1228	[1,2]
b-CO_3^{2-}<br>Bidentate carbonate	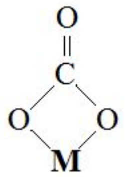	1600, 1348	[1,3]
p-CO_3^{2-}<br>Polydentate carbonate	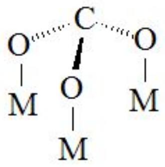	1420, 1445	[1,3]
COOH<br>Carboxylate	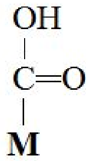	1387	[2]
i-CO_3^{2-}<br>Ionic carbonate		1432	[2]

[1] Freitas, I. C., Damyanova, S., Oliveira, D. C., Marques, C. M. P., & Bueno, J. M. C. (2014). Effect of Cu content on the surface and catalytic properties of Cu/ZrO2 catalyst for ethanol dehydrogenation. *Journal of Molecular Catalysis A: Chemical*, *381*, 26-37.

[2] Knapp, R., Wyrzgol, S. A., Jentys, A., & Lercher, J. A. (2010). Water–gas shift catalysts based on ionic liquid mediated supported Cu nanoparticles. *Journal of Catalysis*, *276*(2), 280-291.

[3] Bachiller-Baeza, B., Rodriguez-Ramos, I., & Guerrero-Ruiz, A. (1998). Interaction of carbon dioxide with the surface of zirconia polymorphs. *Langmuir*, *14*(13), 3556-3564.
